# Acute increases in brain-derived neurotrophic factor in plasma following physical exercise relates to subsequent learning in older adults

**DOI:** 10.1038/s41598-020-60124-0

**Published:** 2020-03-10

**Authors:** Jonna Nilsson, Örjan Ekblom, Maria Ekblom, Alexander Lebedev, Olga Tarassova, Marcus Moberg, Martin Lövdén

**Affiliations:** 1 Aging Research Center, Karolinska Institutet and Stockholm University, Stockholm, Sweden; 2 Swedish School of Sport and Health Sciences, Stockholm, Sweden; 3 Department of Clinical Neuroscience, Karolinska Institutet, Stockholm, Sweden

**Keywords:** Cognitive ageing, Learning and memory

## Abstract

Multidomain lifestyle interventions represents a promising strategy to counteract cognitive decline in older age. Brain-derived neurotrophic factor (BDNF) is essential for experience-dependent plasticity and increases following physical exercise, suggesting that physical exercise may facilitate subsequent learning. In a randomized-controlled trial, healthy older adults (65–75 years) completed a 12-week behavioral intervention that involved either physical exercise immediately before cognitive training (*n* = 25; 13 females), physical exercise immediately after cognitive training (*n* = 24; 11 females), physical exercise only (*n* = 27; 15 females), or cognitive training only (*n* = 21; 12 females). We hypothesized that cognition would benefit more from cognitive training when preceded as opposed to followed by physical exercise and that the relationship between exercise-induced increases in peripheral BDNF and cognitive training outcome would be greater when cognitive training is preceded by physical exercise. Greater increases of plasma BDNF were associated with greater cognitive training gains on trained task paradigms, but only when such increases preceded cognitive training (ß = 0.14, 95% CI [0.04, 0.25]). Average cognitive training outcome did not differ depending on intervention order (ß = 0.05, 95% CI [−0.10, 0.20]). The study provides the first empirical support for a time-critical but advantageous role for post-exercise increases in peripheral BDNF for learning at an interindividual level in older adults, with implications for future multidomain lifestyle interventions.

## Introduction

Late-life cognitive impairment and dementia have increasingly serious human, social, and economic burdens, and prevention is a key element to counteract this development^1^. Epidemiological studies have found that cognitive performance across the lifespan is associated with various life-style factors, including education^2^, occupational complexity^3^, and physical activity^4^. Single-domain intervention studies have provided weak support for an advantageous role of cognitive training and physical exercise for cognition in older age, but effects tend to be small and inconsistent^5–8^. It has consequently been suggested that multidomain interventions that target several lifestyle factors may be needed for optimal preventative effects^9^. Mechanistic accounts for how such multidomain interventions should be designed to optimize effects are, however, largely lacking. Brain-derived neurotrophic factor (BDNF) is a neurotrophin that is essential for neuronal plasticity^10,11^ and increases transiently from physical exercise^12–14^. BDNF can therefore be expected to be of mechanistic relevance for the interactive effects of physical exercise and cognitive training on cognition. Here we investigate the cognitive effects of a 12-week multidomain intervention comprising cognitive training and physical exercise, in close temporal succession, in healthy older adults, focusing on BDNF as a possible biological mechanism.

Under conditions of physical exercise, the brain is thought to be one of the primary tissues to produce and release BDNF into the blood circulation via the blood-brain barrier^15,16^. Peripheral BDNF has been found to correspond moderately to highly with cortical BDNF in rodents and pigs, which supports the use of peripheral BDNF to gage information about the otherwise inaccessible central BDNF in humans^17,18^. The vast majority of peripheral BDNF is bound to platelets and only a small fraction is freely circulating in plasma, of which only the latter is immediately bioavailable^19–21^. It is well established that acute exercise is a potent stimulus for transiently increasing peripheral BDNF concentrations in serum as well as in plasma^12–14^. Whilst baseline peripheral BDNF levels tend to decrease with age^22,23^, acute exercise-related increases do not appear to be affected by age^12,14^, with reliable increases being reported also in older age groups^24^. Physical exercise therefore represents a potential route by which BDNF and its presumed neuroplastic consequences can be acutely enhanced for the benefit cognition in old age.

The potentially facilitating effect of physical activity on neuroplastic processes suggests that combining physical and cognitive training interventions may result in additive effects on cognition^21,25,26^. A recent meta-analysis of 41 studies investigated the effect of such dual interventions in older adults and concluded that there was no additive effect when comparing the combined intervention to cognitive training alone^27^. Larger effect sizes were, however, demonstrated for studies that implemented physical exercise and cognitive training simultaneously (e.g. exergames, dance) compared to studies that conducted the interventions in separate sessions, suggesting that the null finding may be due to suboptimal timing of the interventions^27,28^. The temporary nature of the exercise-induced increase in peripheral BDNF concentrations, with a return to baseline 10–60 min after the end of physical activity, suggest that a temporally close succession of cognitive training may be necessary for optimal benefit^13^. Taking such a mechanistic perspective, Walsh *et al*. (2016) proposed that to effectively take advantage of exercise-induced increases of peripheral BDNF, multidomain interventions should be conducted so that the cognitive engagement immediately follows physical exercise cessation.

The primary aim of the present study was therefore to directly investigate the effect of timing in a combinatory intervention of cognitive training and physical exercise in older adults. To this end, participants were randomized to engage in cognitive training before or after physical exercise, with immediate succession from one intervention to the next in each session, during a 12-week long intervention period. Acute changes in BDNF concentrations in response to the physical exercise and cognitive training were assessed at pretest, in serum and in plasma. Given the temporary nature of the exercise-induced BDNF increase and its presumed neuroplastic effects, we hypothesized that cognition in older adults would benefit more from cognitive training when it is preceded as opposed to followed by physical exercise. In other words, we hypothesized that by administering physical exercise before cognitive training, the transient BDNF increase would coincide with and facilitate cognitive training outcome. Clarifying this is important for informing how physical training should best be combined with cognitive training for combatting late-life cognitive decline. On the mechanistic role of BDNF, it was hypothesized that the relationship between exercise-induced increases in peripheral BDNF and cognitive training outcome would be greater when cognitive training is preceded as opposed to followed by physical exercise, establishing a role for exercise-induced increases in BDNF for facilitating learning in older adults. Compared to cognitive training and physical exercise conducted as single-domain interventions, it was furthermore hypothesized that a combined intervention would provide a greater benefit to cognition, independent of order. The study has been pre-registered at the Open Science Framework (https://osf.io/wfgr4, 13/02/2018) and retrospectively registered as a clinical trial at ISRCTN (ISRCTN13543922, 18/09/2019).

## Methodology

### Participants

Healthy participants between 65 and 75 years were recruited and following an initial screening of study criteria, participants were invited to an information meeting at which detailed study information was given, study criteria confirmed and informed consent obtained. The study criteria were specified to ensure that participants were free of any serious physiological or psychological illness and were able to complete all aspects of the study, with a medical telephone interview ensuring suitability for the fitness tests (for complete list of study criteria, see S1). No requirements were imposed in regards to participants’ physical fitness or activity level, except that participants had to be able to maintain their previous level of physical activity, in addition to any study intervention. The study was approved by the ethical review board in Stockholm (Regionala Etikprövningsnämnden, Stockholm, case number 2017/1115-31/4) and conducted in accordance with the Declaration of Helsinki.

### Design and procedure

**Figure 1:**
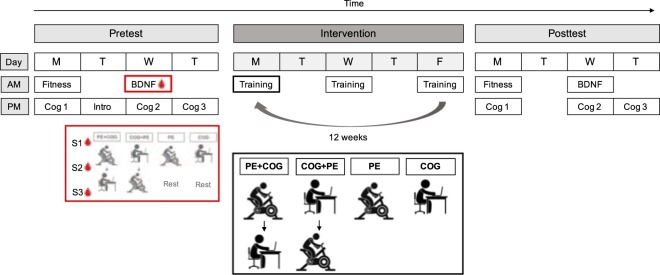
Schematic of the study procedure. Pretest and posttest included physical fitness tests (Fitness) and assessment of acute changes in BDNF concentrations (BDNF), both performed in the morning (AM), as well as an extensive cognitive assessment completed over three afternoon sessions (Cog 1–3; PM). The pretest phase additionally included an introduction to the allocated training intervention (Intro). At the BDNF assessment, the allocated intervention was performed and three blood samples were obtained: before (S1) and after (S2) the first intervention and after the second intervention or rest (S3; see red inset). During the intervention phase, training sessions took place every second weekday (2–3 sessions/week), according to the allocated intervention: physical exercise followed by cognitive training (PE + COG), cognitive training followed by physical exercise (COG + PE), physical exercise only (PE) and cognitive training only (COG). Note that an accelerometer assessment was also conducted (not depicted) and that only the BDNF assessment at pretest was considered in the present study.

The study procedure consisted of a pretest phase, an intervention phase and a posttest phase, with random assignment to intervention (1:1): physical exercise before cognitive training (PE + COG), cognitive training before physical exercise (COG + PE), cognitive training only (COG) or physical exercise only (PE; Fig. 1). Age, physical activity level (self-reported time spent in vigorous and moderate physical activity in a typical week; score range 0–5) and general cognitive performance (matrix reasoning performance; score range 0–18) were used as stratifiers in the randomization. Randomization was conducted using label shuffling with post-hoc non-parametric tests for the stratifiers and was run separately in rounds of approximately 24–32 participants in the R programming environment (version 3.5.1^29^; script available on request). Randomization was headed by A.L., who was not involved in participant enrollment or data collection. Whilst participants were not blinded to the existence of four intervention arms, they were blinded to the hypotheses concerning differential cognitive outcome from the interventions.

The intervention phase lasted for 12 weeks with training visits occurring every second week day (2–3 sessions/week on alternating weeks; Fig. 1). The allocated intervention was also performed as part of the BDNF assessment at pretest and posttest, resulting in a maximum of 32 training visits. All training sessions started with 15 minutes of seated rest, which aimed to limit previous physical activity as a potential source of variation. Training sessions were conducted in groups of maximum six participants and took place between 08:00 and 12:30, with training time being kept constant for each participant throughout the intervention. Training time was counterbalanced so that each training session started on average at the same time in the four intervention groups. The pretest and the posttest phase were completed in the week before and after the intervention and included an extensive cognitive assessment, physical fitness tests, accelerometer measurements, and assessment of acute changes of peripheral BDNF in response to the allocated intervention type (Fig. 1). With the exception of the physical fitness tests and the BDNF assessments, which were conducted at the Swedish School of Sports and Health Sciences (Stockholm, Sweden), all study visits were conducted at Karolinska Institute (Stockholm, Sweden).

#### Cognitive training

The adaptive cognitive training program targeted the working memory construct of updating. In each session, participants trained on an n-back task and a running span task for approximately 30 minutes, both of which require continuous maintenance and updating of mental representations (S2). Two blocks of n-back (6 runs/block) were alternated with two blocks of running span (2 runs/block), alternating between which task that was presented first from session to session. Whenever performance in a run reached a fixed criterion, task difficulty was increased by imposing a greater working memory load (S2). Improvements in processing efficiency over strategy-based processes were promoted by the inclusion of the two updating tasks (n-back, running span) as well as four different stimuli sets for each task type (S2). Participants also completed an additional n-back task at the end of each cognitive training session, which was identical across the study period (i.e. non-adaptive), which allowed for performance during the intervention to be compared across participants (duration = 5 min, max score = 24; S3).

#### Physical exercise

Each aerobic exercise session lasted for approximately 35 minutes, of which 5 minutes were spent warming up and 30 minutes in moderate aerobic activity at a heart rate corresponding to 65–75% of participants’ individual maximum heart rate (HR_max_), as indicated by the fitness assessment. The intensity was systematically varied in six 5-minute intervals, varying heart rate from 65%, 70%, 75%, 70%, 75%, and 65% of HR_max_ during the respective intervals. The first three sessions constituted warm-in sessions, in which the 5-minute intervals instead alternated between 65% and 70% of HR_max_. Heart rate was measured using a chest-strap heart rate monitor Polar monitor (m400, Polar Electro Oy, Kempele, Finland) and was displayed next to an individualized intensity interval card on the bike, allowing for continuous self-monitoring. At the end of each interval, heart rate and rated perceived exertion (RPE; Borg., 1970) were recorded by the test leader, who also advised on how to appropriately reach and maintain the indicated heart rate. If RPE was outside the range of 13 (“somewhat hard”) to 15 (“hard”), participants were instructed to increase or reduce the intensity accordingly. The exercise intensity was thereby intended to allow for possible improvements in fitness over the training period. Videos of cycling rides in the Stockholm area were displayed on a large screen to improve motivation.

#### Combined physical exercise and cognitive training

In the PE + COG group, participants were allowed a brief moment to have some water and to put on a sweater before sitting down at the computer, at which a 1-minute countdown passed before the cognitive training started. In the COG + PE group, participants moved to the exercise bikes and started the warm-up as soon as the cognitive training session had ended.

#### Cognitive assessment

The cognitive assessment consisted of 19 tests, of which 18 were intended for measuring eight cognitive constructs of interests in the present study (S4). The cognitive constructs were selected to systematically vary in similarity to the cognitive training tasks: trained working memory tasks (trained stimuli), trained working memory tasks (untrained stimuli), untrained working memory tasks (updating, switching) and untrained cognitive domains (episodic memory, processing speed, spatial reasoning and verbal reasoning; S4). The trained working memory tasks (trained stimuli) and (untrained stimuli) used the same n-back and running span tasks as in the cognitive training, but differed in that the former used the same stimuli as in the training whilst the latter used novel stimuli. The cognitive tests were completed over three afternoon sessions, in the same order for all participants, at pretest and posttest.

#### Fitness

Participant’s cardiovascular fitness was assessed using a maximal treadmill ergometer test, preceded by a submaximal cycle ergometer test, in the first half of the day (08:30–12:30), with all participants being tested individually and at the same time at pretest and posttest. The submaximal test ensured that exercise intensity for the exercise intervention could be determined also for those who were unable to complete the maximal test. Heart rate (HR) was measured using the same sensor as during the intervention.

The submaximal cycle ergometer test was performed on a cycle ergometer (model 828E, Monark, Varberg, Sweden) prior to the maximal test. The Ekblom-Bak test (EB-test) protocol was used^30^ including two steps: 4 minutes cycling at a standard work rate (resistance of 0.5 kp, cadence of 60 revolutions per minute) and 4–5 minutes cycling at a higher work rate (50–70% of maximal capacity, determined individually by a steady state HR of 110–140 bpm and RPE of around but not above 16). Mean HR was calculated from the last minute on each work rate. Maximum rate of oxygen consumption (VO_2_) max was estimated using sex-specific prediction equations based on data from EB-test, based on HR at standard work rate, a factor for the higher work rate, change in HR between the standard work rate and the higher work rate, age and sex^30^.

Maximum rate of oxygen consumption (VO_2_) max was measured directly from the maximal treadmill ergometer test. After a 5–10 minutes warm-up, the maximal test started with an initial treadmill incline of 1 degree at a comfortable speed (around RPE 12–13) and continued with increases of incline and/or speed every minute until volitional exhaustion. Participants wore a harness attached to the ceiling to protect from falls and to increase confidence and thereby maximize the chances of reaching volitional exhaustion. VO_2_ max was measured using a computerized metabolic system (Jaeger Oxycon Pro, Hoechberg, Germany). Similar to previous studies^30,31^, the VO2max measurement was accepted if a minimum of three out of five following criteria were achieved: (a) VO_2_ was leveling off despite an increase in speed or decline, (b) RPE reached above 16, (c) a respiratory exchange ratio reached value greater than 1.1, (d) maximal HR within ±15 beats per minute from age-predicted maximal HR and (e) a work with time above 6 minutes was performed. The highest 30 seconds of registered values of VO_2_ and heart rate were referred to as VO_2_ max and HR_max_, respectively. VO_2_ max were expressed in relative (mL kg-1 min-1) units and HR_max_ in beats per minute (bpm). Only the VO_2_ max, and not the submaximal result, were used in the hypotheses testing.

#### BDNF

The blood sampling protocol assessed acute changes in BDNF concentrations in response to the interventions at pretest, at least 48 hours after the fitness assessment. Note that the BDNF assessment was repeated at posttest but was not considered here. To make the assessment representative of a typical training session, the session was always scheduled for the first half of the day (08:00–12:30), at a similar time as the individual participant’s intervention sessions. Upon arrival, a peripheral intravenous catheter was inserted into the antecubical vein to facilitate timely blood sampling. After 15 minutes of seated rest, the first sample was drawn. Participants subsequently commenced with cognitive training or physical exercise, depending on the allocated intervention, after which the second sample was immediately collected. Participants then engaged in cognitive training, physical exercise or seated rest, also depending on intervention. after which the third sample was drawn. To avoid contamination between samples, the catheter was flushed with saline solution between each draw. For each sample, 10 mL was collected into (a) heparinized containers (plasma) and (b) sterile separator tubes (serum). The blood sampled in the heparinized container were spun at 4 °C for 3 min at 6000 rpm to separate the plasma, which was placed in a new container and frozen at −80 °C until analysis. The blood sampled in the additive-free container was left for 30 minutes to clot after which it was spun at 4 °C for 15 min at 6000 rpm to separate the serum, which was then placed in a new container and frozen at −80 °C until analysis. BDNF concentrations were quantified using an enzyme-linked immunosorbant assay according to the manufacturer’s instructions (Human BDNF Quantikine Immunoassay, DBD00, R & D Systems).

#### Accelerometry

The accelerometry measures assessed whether participants in the different intervention groups differed in regards to compensatory changes in physical activity outside the study context. Since the accelerometers primarily capture locomotion activity and underestimate non-ambulatory activities, such as cycling, the accelerometer assessment primarily reflected physical activity outside the physical exercise intervention. The accelerometers, which were lightweight and worn in an elastic elastic band on the hip during the day (Actigraph GT3X+, Actigraph LCC; Pensacola, FL, USA), were worn for 7 days before the first study visit, reflecting participants’ usual pattern of movement, and for the last 7 days of the intervention, reflecting potential changes to the pattern of movement due to intervention engagement. Accelerometer data were recorded as raw data (sample rate set to 30 Hz) from all three axes, which were combined into a resulting vector, and extracted as 60 seconds epoch using a low frequency extension filter.

### Statistical analysis

#### Cognitive composites

Cognitive composites were created by taking the unit-weighted average of the two or three relevant measures at pretest and posttest (S4). Composite scores were subsequently standardized by pretest performance ((x-mean_pretest_)/sd_pretest_) and converted into T scores (mean_pretest_ = 50, sd_pretest_ = 10; S5). All cognitive composite scores were approximately normally distributed (skewness < 3.0, kurtosis < 10.0). Cognitive composites could be calculated at pretest and posttest for all 97 participants, with the exception of the episodic memory composite for one participant in the COG group and one in the PE + COG group at posttest.

#### BDNF

Concentrations in plasma were available for all 97 participants for all three samples.

Four serum samples were not handled according to protocol and were therefore excluded from analyses (sample 1 for one participant in the COG + PE group, samples 1–3 for one participant in the COG group and one in the PE group), resulting in available BDNF concentrations in serum for 94 participants for sample 1 and for 95 participants for sample 2 and 3. Serum concentrations were normally distributed at the three sampling timepoints (skewness < 3.0, kurtosis < 10.0), with no extreme outliers (outlier labelling rule, IQR = 3.0). In contrast, plasma concentrations were severely negatively skewed at all timepoints (skewness > 3.0, kurtosis > 10.0). Natural log-transformations were therefore performed, which were successful in making the distributions normal at all timepoints, with no extreme outliers being subsequently detected. To arrive at a measure of acute BDNF change following physical exercise, the concentration measured before exercise was simply subtracted from the concentration measured immediately after. For groups with exercise as their first intervention (PE, PE + COG), sample 1 was therefore subtracted from sample 2, and for the group with exercise as their second intervention (COG + PE), sample 2 was subtracted from sample 3.

#### Fitness

95 participants successfully completed the maximal treadmill test at pretest and 94 at posttest. Following exclusions made for factors that may have influenced the measurements (e.g. current or residual infection), 94 participants were left with VO_2_ max measurements at pretest (COG = 21, PE = 25, COG + PE = 23, PE + COG = 24) and 89 at posttest (COG = 21, PE = 24, COG + PE = 21, PE + COG = 23). Exercise intensity intervals for the intervention were based VO_2_ max estimates from the submaximal test when direct measurements from the maximum test were unavailable (n = 3). Only direct measurements of VO_2_ max from the maximum fitness test were included for hypothesis testing.

#### Accelerometry

Using standard definitions, sedentary behaviours was defined as <200 counts per minute (cpm) and moderate-to-vigorous physical activity as cpm ≥2690^32^. Proportion of time spent between 06:00 and 23:00 in the different intensities constituted the measures of interest. A minimum of 600 minutes of valid daily wear time for at least 4 days was required to be included in the analyses^33^. Sufficient accelerometery data was available for 95 participants at pretest (COG = 20, PE = 27, COG + PE = 23, PE + COG = 25) and 90 participants at posttest (COG = 17, PE = 26, COG + PE = 23, PE + COG = 24).

#### Linear mixed-effect models

Linear mixed-effects models (LMM) were fitted using the lme4 package (version 1.1-18-1^34^) in the R programming environment (version 3.5.1^29^) employing restricted maximum likelihood (REML) to estimate the parameters. Inferential analyses were performed using the lmerTest package (version 3.1-0^35^) using the Satterthwaite’s approximation to estimate denominator degrees of freedom for *F* statistic and to obtain p-values (type III analysis of variance). All possible interactions between fixed factors were included in all models. For analyses concerned with change in cognition from pretest to posttest (cognitive composites), intercept for subject was entered as a random factor. In analyses on change in performance across all cognitive training visits (non-adaptive n-back), intercept and linear slope were entered as random factors. In analyses that included serum concentrations and cognitive composites, serum concentrations were divided by 1000 to achieve more compatible scales.

Correction for testing multiple models was based on the number of models used to test a single hypothesis, using Bonferroni-corrected α-levels. For example, when a hypothesis concerning BDNF was tested in serum and in plasma, the α-level was corrected for the use of two models to test a single hypothesis (α = 0.05/2 = 0.025). Post-hoc tests were performed to follow up on significant interactions to aid interpretation. Estimated marginal means were contrasted using the emmeans package (version 4.1), employing the Tukey method to adjust for multiple comparisons and Satterthwaite’s approximation for determining degrees of freedom. For contrasting associations, relevant zero-order correlations were computed and compared using Fisher’s r to z transformation. No correction of the α-level was applied for post-hoc analyses.

#### Power calculation

Power calculation techniques for linear mixed modelling are complex with no one agreed-upon solution. Therefore, we opted for a guiding power calculation using the study design for the primary hypothesis using G*Power to provide an approximation of power and required sample size. Specifically, a 2 (time; pretest versus posttest) × 2 (group; physical exercise before working memory training versus working memory training before physical exercise) mixed analysis of variance, based on a small-to-medium effect size of 0.4 standard deviations and an alpha level of 0.05, requires a total sample size of 60 subjects (30 per group) to achieve an acceptable power of 0.8. Based on this approximation, the target sample size per group was set to 30 for all four intervention groups, resulting in a total target sample size of 120.

## Results

Results are reported in the following order: descriptive results, results from confirmatory analyses relating to the pre-registered hypotheses and analysis plan (H1-H7; osf.io/9m6aj;) and selected exploratory analyses. For the purpose of brevity, some descriptive and inferential statistics are included in supplementary materials (S6–S11).

### Descriptive results

#### Study sample

**Figure 2:**
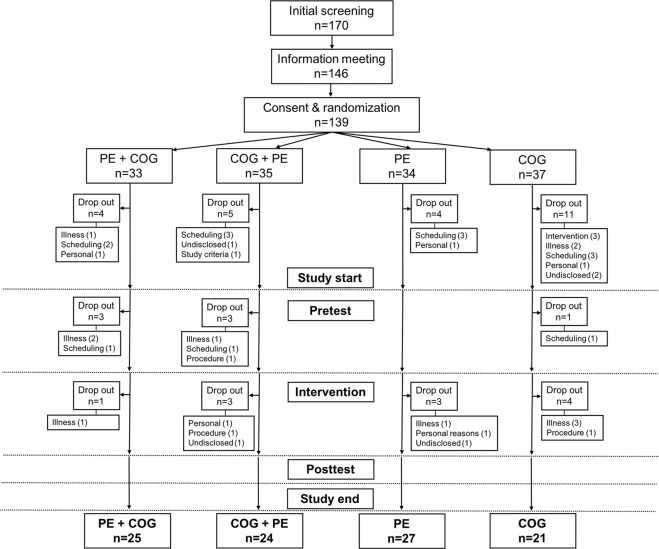
Recruitment diagram. Number of subjects initially screened, attending the information meeting, and subsequently consenting and being randomized into the study and completing each of the four intervention programs (PE + COG, COG + PE, PE, COG). Number of drop-outs is specified for each phase of the study procedure and reasons discontinuing the study are specified in brief (drop-out analysis, S6). Illness = illness, injury or health reasons. Scheduling = inability to adhere to the study schedule. Personal = personal reasons. Undisclosed = reason not specified. Study criteria = one or more study criteria no longer fulfilled. Intervention = dissatisfied with allocated intervention. Procedure = dissatisfied with one or more aspects of the study procedure.

139 participants were randomized, of which 97 completed the study and were included in analyses (Fig. 2). The majority of drop-outs occurred before the first study visit, with a similar number of drop-outs (*n* = 3–6) for the intervention groups after study start. The effects of dropout were small with no evident differential effects on age, reasoning ability and self-reported physical activity in the different intervention groups (S6). Participants in the four intervention groups were comparable at baseline (Table 1). Data collection for the study commenced in September 2017 and was completed in July 2018.

**Table 1:** Baseline demographic, physiological and cognitive information.

		COG			PE			COG + PE			PE + COG		
		*n*	*mean*	*sd*	*n*	*mean*	*sd*	*n*	*mean*	*sd*	*n*	*mean*	*sd*
*Demographic*	Age (years)	21	70.95	3.04	27	70.30	3.00	24	70.29	3.11	25	70.28	2.70
	Education (level)	21	2.71	0.64	27	2.52	0.80	23	2.70	0.56	25	2.68	0.56
	Sex (females/males)	21	12/9	—	27	15/12	—	24	11/13	—	25	13/12	—
*Physiology*	BMI (kg/m^2^)	21	25.21	3.45	26	26.23	3.17	22	26.98	4.34	25	25.05	3.69
	VO2 max (mL/kg/min)	21	31.01	5.00	25	30.78	5.11	23	31.00	6.03	25	33.07	5.71
	Max heart rate (beats/min)	21	167.60	10.03	27	161.50	15.39	24	164.90	11.16	25	162.20	12.42
*Physical act*.	Sedentary activity (%)	20	57.00	10.00	27	55.00	9.00	23	56.00	8.00	25	56.00	6.00
	Moderate-vig. activity (%)	20	6.00	3.00	27	6.00	2.00	23	6.00	2.00	25	7.00	3.00
*Cognition*	MMSE (score)	21	29.24	0.83	27	29.52	0.75	24	29.33	1.05	25	29.32	1.15
	Reasoning (score)	21	5.86	2.95	27	6.04	2.77	24	6.50	3.07	25	6.16	2.48

VO_2_ max represents direct measurements derived from maximal treadmill test, which was available for 94 participants. Maximal heart rate was derived from the maximal treadmill test where available (n = 94) and otherwise from the submaximal ergometer test (n = 3). Physical activity measures were derived from the accelerometry assessment and represent percentage time spent in sedentary and moderate-vigorous activity between 06:00 and 23:00, averaged across the 7-day accelerometry assessment at pretest. Education level: 1 = elementary school, 2 = high school, 3 = university. Mini Mental State Exam: maximum score 30. BMI = body max index. VO_2_ max = maximum rate of oxygen consumption. MMSE = Mini mental state exam (max score 30, <26 excluded). Reasoning = progressive matrix reasoning (max score 18). PE = physical exercise. COG = cognitive training.

#### Attendance

The intervention groups attended the same number of training sessions, *F*(1,95) = 0.256, *p* = 0.614, with the PE + COG group attending 24.48 sessions (SD = 2.02), the COG + PE group 25.17 sessions (SD = 2.46), the COG group 25.05 sessions (SD = 2,50) and the PE group 24.63 sessions (SD = 2.71).

#### Accelerometry

Participants engaged in more prolonged sedentary bouts in the last week of the intervention (M = 58.3%, SD = 8.10) compared to the week before study start (M = 55.9%, SD = 8.47), *F*(1, 91.38) = 6.069, *p* = 0.0156, but the four intervention groups did not differ in overall, *F*(1, 175.12) = 0.1856, *p* = 0.667, or in change of sedentary behavior, *F*(1, 90.95) = 1.168, *p* = 0.283. Participants spent 6.40% (SD = 2.50) of their time in moderate-to-vigorous physical activity prior to study start and this did not change as a result of the intervention, *F*(1, 90.38) = 0.970, *p* = 0.327. The intervention groups did also not differ in overall, *F*(1, 158.90) = 0.027, *p* = 0.869, or in change of moderate-to-vigorous activity, *F*(1, 90.80) = 1.167, *p* = 0.283.

#### BDNF assessment

Mean resting BDNF concentration at pretest was 24673 pg/mL in serum (*SD* = 5600), ranging from 11598 to 46013. Median resting concentration was 141 pg/mL in plasma (IQR = 193), ranging from 28 to 6820 pg/mL. The average intensity of physical exercise during the BDNF assessment was 72.4% of HR_max_, with no significant difference between groups (PE, PE + COG, COG + PE), *F*(1,73) = 0.308, *p* = 0.58.

### Confirmatory analyses

#### The acute change in peripheral BDNF levels in older adults will be greater immediately following physical exercise compared to immediately following working memory training (H5)

**Figure 3:**
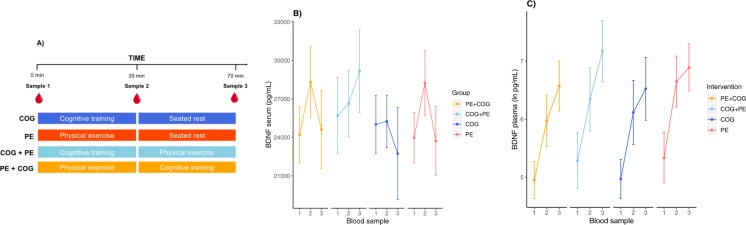
Blood sampling protocol (**A**) and acute BDNF changes in response to the interventions in serum (**B**) and in plasma. (**C**) BDNF concentrations in serum increase in response to physical exercise (sample 1 to 2 in PE and PE + COG groups; sample 2 to 3 in COG + PE group) but not in response to cognitive training (sample 1 to 2 in COG and COG + PE; sample 2 to 3 in PE + COG group). BDNF concentrations in plasma increase independent of intervention. Note that the hypothesis testing involved specific group comparisons and merging of groups. PE = physical exercise, COG = cognitive training.

For serum, there was a significant interaction between time (sample 1 vs. sample 2) and intervention (PE & PE + COG vs. COG & COG + PE), *F*(1, 92.3) = 26.6, *p* < 0.0001, and a significant effect of time, *F*(1, 92.3) = 50.65, p < 0.0001 (*α*_*corr*_ = 0.025). Post-hoc pairwise comparisons revealed that BDNF concentrations in serum increased significantly from sample 1 to sample 2 following PE, *t*(92.2) = −9.07, *p* < 0.0001, but not following COG, t(92.4) = −1.331, *p* = 0.1866, in support of the hypothesis. For plasma, there was no interaction between time and intervention, *F*(1, 94.8) = 0.0057, *p* < 0.9398, but a significant effect of time, *F*(1, 94.8) = 117.2872, *p* < 0.0001, demonstrating a reliable increase from sample 1 to sample 2 across groups, t(94.8) = −10.801, p < 0.0001 (*α*_*corr*_ = 0.025). The acute mean change in BDNF concentrations are visualized for all intervention groups in Fig. 3.

In descriptive terms, following physical exercise, mean serum concentrations increased from 23964 pg/mL (*SD* = 4979) to 28244 pg/mL (*SD* = 6365), reflecting an average 18% change, whilst non-transformed median plasma concentrations increased from 192 pg/mL (*IQR* = 352) to 802 pg/mL (*IQR* = 898), reflecting an increase of over 300% (S7). Following cognitive training, mean serum concentrations demonstrated virtually no average change from 25011 pg/mL (*SD* = 4958) to 25238 pg/mL (*SD* = 4510), whilst non-transformed median plasma concentrations increased from 129 pg/mL (*IQR* = 156) to 388 pg/mL (*IQR* = 510), reflecting a 200% increase (S7).

#### The acute changes in peripheral BDNF levels in older adults following physical exercise and cognitive training will remain after 30 minutes (H6)

For serum, there was no statistically significant interaction between intervention (COG vs. PE) and time (sample 2 vs. sample 3), *F*(1, 44) = 1.8011, *p* = 0.1865, but a significant effect of time, *F*(1, 44) = 22.0243, *p* < 0.0001, reflecting a decrease from sample 2 to sample 3 across groups, *t*(44) = 4.639, *p* < 0.0001 (*α*_*corr*_ = 0.025). For plasma, there was no significant interaction between group and time, *F*(1, 46) = 0.5877, *p* = 0.4472, but a significant effect of time, *F*(1, 46) = 9.1133, *p* = 0.0041, reflecting an increase from sample 2 to sample 3 across groups, *t*(46) = −3.019, *p* < 0.0041 (*α*_*corr*_ = 0.025). Thus, in contradiction of the hypothesis, BDNF concentrations decreased in serum and increased in plasma during rest, independent of the prior intervention (Fig. 3).

#### Moderate intensity physical exercise 2–3 times per week over 12 weeks improves physical fitness in older adult (H7)

There was no significant interaction between intervention group (PE + COG vs. COG + PE vs. COG vs. PE) and time (pretest vs. posttest), *F*(1,87.1)=0.47, *p* = 0.49, and no main effect of time, *F*(1,86.3) = 1.07, *p* = 0.30, or group, *F*(1,91.7) = 2.23, *p* = 0.14, evidencing no differential improvement in cardiovascular fitness in the different intervention groups.

#### Cognition in older adults benefits more from repeated sessions of working memory training when each training session is combined with physical exercise, irrespective of order, compared to working memory training alone (H3)

**Figure 4:**
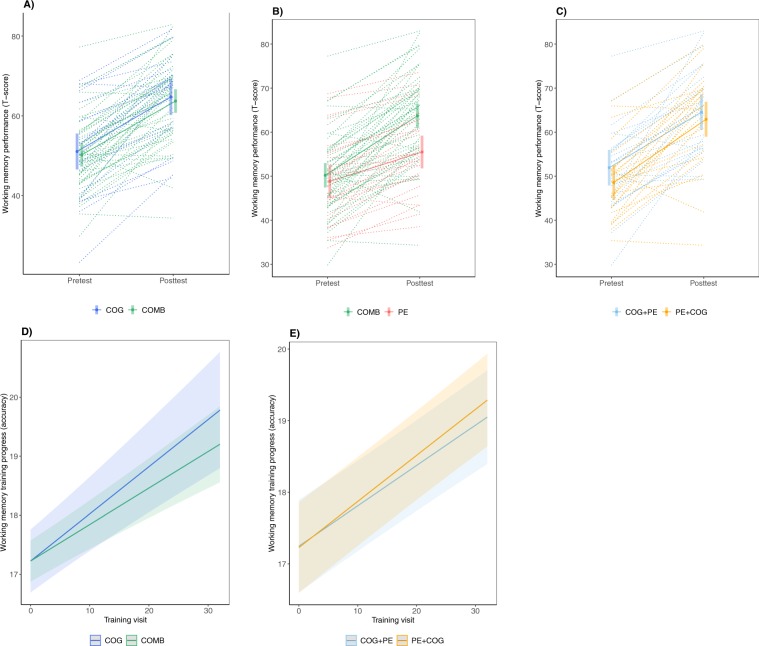
Change in working memory performance from pretest to posttest (**A**–**C**) and across cognitive training visits. (**D**,**E**) Estimated marginal means (error bars = 95% confidence intervals) derived from linear mixed models testing intervention effects on performance in trained tasks (trained stimuli), in the context of hypothesis 3 (COG + PE & PE + COG vs. COG; **A**), hypothesis 4 (COG + PE & PE + COG vs. PE; **B**) and hypothesis 1 (COG + PE vs. PE + COG; **C**), with spaghetti lines corresponding to individual change trajectories (T-scores, *M* = 50, *SD* = 10). Model-implied regression lines illustrating the effect of training visit on progress in the n-back task during the cognitive training intervention (maximum score = 24), in the context of hypothesis 3 (COG + PE & PE + COG vs. COG; D) and 4 (COG + PE & PE + COG vs. PE; **E**). COG = cognitive training, PE = physical exercise, COMB = PE and COG, irrespective of order.

No significant interaction was detected between intervention (PE + COG & COG + PE vs. COG) and time (pretest vs. posttest) for any of the cognitive composites (H3a-c; trained working memory in Fig. 4A) nor for training progress (H3d; Fig. 4D), contradicting the hypothesized difference in cognitive outcome between groups that received both cognitive training and physical exercise relative to cognitive training alone (S9). There was a significant main effect of time (pretest vs. posttest) for performance on trained working memory tasks (trained stimuli & untrained stimuli), untrained working memory tasks (updating), and untrained cognitive domains (episodic memory & spatial reasoning), reflecting improved performance at posttest compared to pretest, as well as a significant main effect of training visit (1–32) on n-back performance, reflecting a linear improvement over the course of the cognitive training intervention (S9).

#### Cognition in older adults benefits more from repeated sessions of physical exercise when each exercise session is combined with working memory training, irrespective of order, compared to physical exercise alone (H4)

A significant interaction was detected between intervention (PE + COG & COG + PE vs. PE) and time (pretest vs. posttest) for trained working memory tasks (trained stimuli & untrained stimuli; Fig. 4B; *α*_*corr*_ = 0.025), reflecting greater improvements for the group that was exposed to the cognitive training (PE + COG & COG + PE) relative to the group that was not (PE; S10). There was no statistically significant intervention-by-time interaction for any other cognitive composite, providing no evidence that the cognitive training resulted in improvements beyond the trained tasks. There was a significant main effect of time (pretest, posttest) for the same cognitive composites as for H3, reflecting a general improvement from posttest to pretest, as well as a significant main effect of training visit (1–32) on n-back performance, reflecting a linear improvement across the cognitive training intervention (S10).

#### Cognition in older adults benefits more from repeated sessions of working memory training when each training session is directly preceded as opposed to followed by physical exercise (H1)

No significant interaction was detected between intervention (COG + PE vs. PE + COG) and time (pretest vs. posttest) for any of the cognitive composites (H1a-c; trained working memory in Fig. 4C) nor on training progress (H1d; Fig. 4E), contradicting the hypothesized difference in cognitive outcome when cognitive training is preceded as opposed to followed by physical exercise (S8). There was a significant main effect of time (pretest, posttest) for the same cognitive composites as for H3 and H4, reflecting a general improvement from posttest to pretest, as well as a significant main effect of training visit (1–32) on n-back performance, reflecting a linear improvement across the cognitive training intervention.

#### The relationship between change in peripheral BDNF levels in response to physical exercise at pretest and the outcome of repeated sessions of working memory training is greater when each training session is directly preceded as opposed to followed by physical exercise (H2)

**Figure 5:**
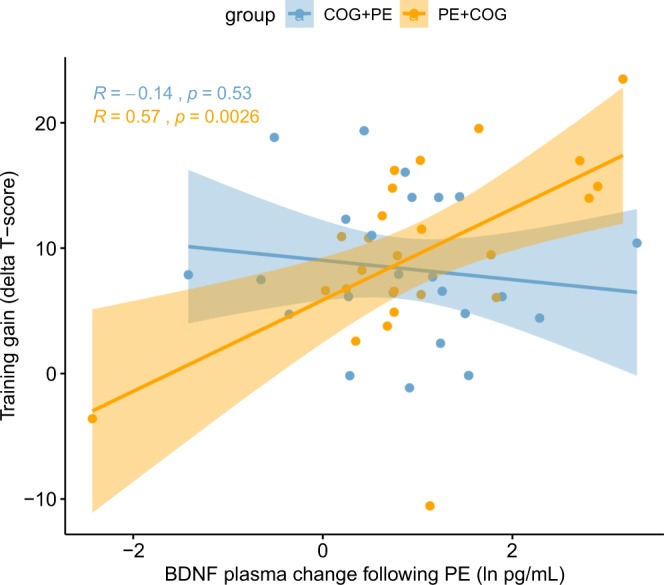
Visualization of the differential relationship between acute BDNF change and pre-post change in trained task performance in the COG + PE and PE + COG groups. Scatterplot with individual data points and zero-order correlations between training gains (pre-post change in performance on trained tasks with untrained stimuli) and acute change in BDNF concentrations in response to physical exercise in plasma at pretest (R). A relationship between exercise-induced BDNF change and cognitive training gain only exists in the group that received physical exercise prior to cognitive training (COG + PE, pale blue). Note that the three-way interaction remains significant at the corrected *α*-level even when the two potential outliers in the PE + COG group are excluded, *F*(1,43) = 7.20, *p* = 0.010. The zero-order correlations also still differed significantly after such outlier removal, Fisher’s *Z* = 2.67, *p* = 0.008 (two-tailed).

In support of the hypothesis, a significant three-way interaction between group (PE + COG vs. COG + PE), time (pretest vs. posttest) and acute changes in BDNF following physical exercise at pretest was found in plasma for performance on trained tasks with untrained stimuli (Table 2; *α*_*corr*_ = 0.0125). Figure 5 visualizes how the relationship between change in performance on trained tasks with untrained stimuli (training gain) and acute BDNF change following physical exercise differed in the two groups. Zero-order correlation coefficients differed significantly, which further supports a differential relationship between training gain and BDNF response in the group that received physical exercise before cognitive training compared to the reverse order, Fisher’s *Z* = 2.63, *p* = 0.009 (two-tailed).

**Table 2:** Statistics for models testing the differential relationship between acute BDNF change and pre-post change in trained task performance in the COG + PE and PE + COG groups.

Factors	*β*	*CI (95%)*		*df (num)*	*df (dn)*	*F*	*p*
**Trained tasks (untrained stimuli)**							
(Intercept)							
Group	−0.1662	−0.4702	0.1378	1	45.00	1.15	0.2896
Time	0.3454	0.2430	0.4479	1	45.00	43.72	**<0.0001**
BDNF	0.3297	0.0972	0.5622	1	45.00	7.73	**0.0080**
Group × Time	10.0734	−0.1758	0.0290	1	45.00	1.97	0.1669
Group × BDNF	0.0634	−0.2442	0.3709	1	45.00	0.16	0.6882
Time × BDNF	0.0938	−0.0102	0.1977	1	45.00	3.13	0.0834
Group × Time × BDNF	0.1441	0.04025	0.2480	1	45.00	7.39	**0.0093**
**Trained tasks (trained stimuli)**							
(Intercept)							
Group	−0.0992	−0.3540	0.1558	1	45.00	0.58	0.4499
Time	0.5000	0.3535	0.6459	1	45.00	44.88	**<0.0001**
BDNF	0.2904	0.0954	0.4855	1	45.00	8.52	**0.0055**
Group × Time	−0.0248	−0.1710	0.1214	1	45.00	0.11	0.7412
Group × BDNF	−0.0482	−0.3062	0.2100	1	45.00	0.13	0.7161
Time × BDNF	0.0944	−0.0539	0.2428	1	45.00	1.56	0.2187
Group × Time × BDNF	0.0841	−0.0643	0.2325	1	45.00	1.23	0.2726

Standardized parameter estimates from the linear mixed effects models and F-tests of main and interaction effects (Type III anova). For unstandardized parameter estimates (S11). P-values that are below the Bonferroni-corrected significance threshold are highlighted in bold (α_corr_ = 0.0125). Group = COG + PE < PE + COG; Time = pretest <posttest; BDNF = change in plasma BDNF concentration following PE at pretest.

No three-way interaction was detected for any of the remaining cognitive composites, in plasma nor in serum, reflecting support for H2a only (trained working memory tasks; Table 2) and not for H2b-c (untrained working memory tasks and untrained cognitive domains; S11). For cognitive training progress (non-adaptive n-back task; H2d), although a trending interaction was detected between time (cognitive training visit 1–32) and acute change in plasma BDNF, *F*(1,44.15) = 5.056, *p* = 0.029 (α_corr_ = 0.025), the groups (COG + PE, PE + COG) did not differ in regards to the strength of this relationship, *F*(1,44.15) = 0.0001, *p* = 0.99096, contradicting the hypothesized differential relationship (S11).

Significant main effects of change in BDNF plasma were noted for performance on trained tasks (trained and untrained stimuli), reflecting a positive relationship between working memory performance and BDNF response, across time and groups (Table 2). A trending main effect of change in BDNF plasma was also detected for performance on untrained working memory tasks, *F*(1,45.0) = 5.04, *p* = 0.030 (α_corr_ = 0.0125; S11). No other main effects or two-way interactions with BDNF concentration change following PE were detected for any other cognitive composite, in plasma nor in serum (S11).

### Exploratory analyses

**Figure 6:**
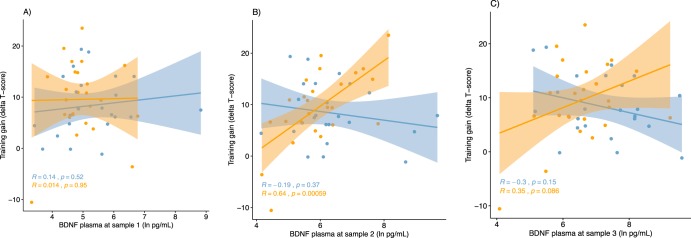
Visualization of the relationship between acute BDNF change and pre-post change in trained task performance in the COG + PE and PE + COG groups, separate for blood sample 1 (**A**), 2 (**B**) and 3 (**C**). Scatterplots visualizing individual data points and zero-order correlations between training gains (pre-post change in performance on trained tasks with untrained stimuli) and acute change in BDNF concentrations in response to physical exercise in plasma at pretest, separate for the PE + COG (orange) and COG + PE (pale blue). BDNF concentrations only predict cognitive training gain in the PE + COG group and only at Sample 2.

An important caveat with the demonstrated support for the hypothesis of a differential BDNF – training gain relationship is that the acute increase in plasma concentrations was not exclusive to physical exercise but occurred also after cognitive training. We therefore explored the three-way interaction effect on BDNF plasma concentrations separately at the three sampling timepoints: before and after the first intervention (samples 1 & 2) and after the second intervention (sample 3). The interaction was significant for sample 2, *F*(1,45.0) = 13.276, *p* < 0.001, and for sample 3, *F*(1,45.0) = 5.564, *p* = 0.023, but not for sample 1, *F*(1,45.0) = 0.289, *p* = 0.594. As visualized in Fig. 6A–C, the zero-order correlation between BDNF plasma concentrations and training gain was only reliable in the PE + COG group at sample 2, at which point the correlation also differed significantly from the COG + PE group, Fisher’s *Z* = 2.58, *p* = 0.001 (two-tailed). The zero-order correlations were not significant in either of the groups at sample 1 and 3, but correlation coefficients differed significantly at sample 3, Fisher’s *Z* = 2.21, *p* = 0.027, but not at sample 1, Fisher’s *Z* = 0.42, *p* = 0.675 (two-tailed). Additionally, in the groups that received only physical exercise or only cognitive training during the intervention, no significant correlations were detected between training gains and BDNF plasma concentrations at any of the sampling timepoints (S12). Thus, BDNF plasma concentrations only appear to predict cognitive training gains when measured immediately after physical exercise and just before cognitive training.

Another important aspect is that evidence for a differential BDNF – training gain association in the PE + COG and COG + PE groups was limited to the cognitive composite that captured performance on trained working memory tasks with *untrained* stimuli. No corresponding evidence for was found for trained working memory tasks with *trained* stimuli, despite equivalent internal consistency reliability for the two composites, as indicated by virtually identical split-half correlations (calculated between odd and even runs/trials at pretest, with Spearman-Brown adjustment) for the composite for trained stimuli (*r*_adj_ = 0.94) and for untrained stimuli (*r*_adj_ = 0.92). To explore this further, the influence of BDNF plasma on training gains were evaluated for each trained test separately. The three-way interaction was again present at sample 2, for running span with trained stimuli, *F*(1,45.0) = 6.3771, *p* = 0.0152, running span with untrained stimuli, *F*(1,45.0) = 6.8023, *p* = 0.01231, n-back with untrained stimuli, *F*(1,45.0) = 5.3366, *p* = 0.02552, but not for n-back with trained stimuli, *F*(1,45.0) = 0.6342, *p* = 0.4300. The three-way interaction was also significant at sample 2 when considering a unit-weighted composite of all four trained tests, *F*(1,45.0) = 4.3407, *p* = 0.0429, indicating that effects may not be exclusive to trained tasks with untrained stimuli.

## Discussion

The present study investigated the effect a 12-week multidomain intervention comprising cognitive training and physical exercise in close temporal succession in older adults, focusing on BDNF as a potential mechanism. Consistent with previous meta-analyses, there was no additive effect of the combined intervention compared to cognitive training alone when the timing of cognitive training and physical exercise was not considered^27,36^. The primary hypothesis concerned the importance of such timing and stated that cognition would benefit more when each cognitive training session is preceded as opposed to followed by physical exercise, consistent with a transient effect of physical exercise on peripheral BDNF and its presumed consequences on neuroplasticity and learning. Contrary to this hypothesis, however, average cognitive outcome did not differ depending on whether participants received physical exercise before or after cognitive training during the intervention. Critically, this was the case even in the presence of reliable increases in peripheral BDNF following physical exercise at pretest, in serum and in plasma, contradicting any average benefit of increased peripheral BDNF post-exercise on subsequent cognitive engagement. At an interindividual level, however, greater increases of plasma BDNF following physical exercise were found to be associated with greater cognitive training gains, but only when such increases preceded cognitive training. This provides the first empirical support for a time-critical but advantageous role of increases in peripheral BDNF for cognitive training outcome in older adults. As such, the study has established that post-exercise increases in plasma BDNF are related to subsequent learning in older adults, with important implications for the design of future multidomain intervention studies that aim to combat the challenge of late-life cognitive impairment.

Consistent with previous research, plasma concentrations were over 100 times lower than serum concentrations at rest, reflecting that the majority of peripheral BDNF is bound to platelets^20^. Resting BDNF concentrations in serum (median = 24409 pg/mL, range = 11598–46013) and in plasma (median = 141 pg/mL, range = 28–6820) corresponded well to previous results in healthy younger adults (20–60 years; n = 140; median_serum_ = 22600 pg/mL, range_serum_ = 1900–51500; median_plasma_ = 92.5 pg/mL, range_plasma_ = 8–927)^23^ and in healthy older adults (70+ years; n = 259; median_serum_ = 21628 pg/mL, SD_serum_ = 10729)^37^. In regards to acute changes, meta-analytic results in younger adults have indicated that exercise-induced BDNF increases are larger in plasma than in serum^12^, with increases in serum ranging from 30% to 100% and in plasma from 30% to 165%^16,38–40^, with some indication of a faster return to baseline in serum compared to plasma^41^. In a previous study similar to this one, a 17% serum increase was reported following 35 minutes of moderate aerobic exercise in older adults^24^. In another study of older adults, a similarly sized serum increase was reported following 40 minutes of moderate aerobic exercise, but no acute changes in plasma were detected prior to an exercise intervention^42^. The present finding of a 18% serum increase therefore appears consistent with previous research whilst the 300% plasma increase must be considered high. Unfortunately, very little is known about exercise-induced increases in plasma and its influences, particularly in older adults, which makes further interpretation of this potential discrepancy difficult.

BDNF in serum increased following physical exercise but not following cognitive training, which is consistent with the hypothesis and previous meta-analytical results^12^. In contrast, plasma BDNF demonstrated a reliable but equivalent increase following both physical exercise and cognitive training. In fact, plasma BDNF increased gradually across the course of the entire session, independent of intervention type. Since the present study did not include a passive control group (no intervention), we cannot exclude the possibility that the test situation itself caused the increases of plasma BDNF. It is conceivable that factors common to all interventions, such as the blood sampling procedure or the unfamiliar environment and test procedure, reflected exposure to non-physical stressors. Mechanistic links have indeed been made that BDNF and glucocorticoids work in conjunction in response to acute stress but little is known about the acute effects of non-physical stressors on peripheral BDNF concentrations in humans^43,44^. Considering previous demonstrations of decreasing or stable resting BDNF concentrations in plasma over the course of the day, we consider diurnal variation in plasma BDNF as a less likely account for the gradual and sizeable increases reported here^45,46^.

Importantly, independent of the cause of the increases of plasma BDNF, it was demonstrated that increases that followed physical exercise were only predictive of training gains when cognitive training followed as opposed to preceded physical exercise. This finding is consistent with the proposed importance of timing in order to effectively take advantage of acute peripheral BDNF changes in this kind of multidomain intervention^28^. In a follow-up analysis, plasma BDNF concentrations were considered separately for each of the three blood samples obtained at pretest (at rest, after 1^st^ intervention and after 2^nd^ intervention). It was shown that plasma BDNF concentrations were only predictive of training gains in the second blood sample and only in the group that received physical exercise prior to cognitive training, which is important for two reasons. First, the lack a relationship with plasma BDNF at rest (baseline) in both groups indicates that the large increases brought about by the first intervention, or the test situation, are important for facilitating subsequent learning. Second, the differential association with plasma BDNF after the first intervention supports that the large increases brought about by the first intervention, or the test situation, are only important for training gains if the cognitive engagement immediately follows such increases. Similarly, follow-up analyses in the groups that received only physical exercise or only cognitive training revealed no relationships with plasma BDNF, indicating that the association is indeed specific to the group that received physical exercise prior to cognitive training. Related to the previous discussion on causal inference, however, the beneficial effect of acute increases in plasma BDNF when it precedes cognitive engagement may not be specific to physical exercise but could be relevant for any factor that increases plasma BDNF. Future research will therefore need to include additional experimental conditions in order to establish whether other, possibly non-physical, events can produce acute increases in plasma BDNF with benefits for subsequent cognition.

In contrast to the demonstrated differential association between plasma BDNF increases and training gains from pretest to posttest by intervention order, no corresponding effect was found for the rate of training progress during the 12-week intervention. The reason for this can only be speculated on but considering that the non-adaptive n-back task was administered after each cognitive training session, over 30 minutes after cessation of the physical exercise, it is possible that the effect of plasma BDNF on cognition was reduced at this point. The finding that BDNF plasma concentrations predicted training gains when measured immediately after physical exercise but not after 30 minutes of cognitive training is consistent with the possibility that any advantageous effect may have faded by the time the non-adaptive n-back task was completed.

It is important to note that the advantageous effect of increases in plasma BDNF following physical exercise for cognitive training outcome was limited to one of the two cognitive composites capturing performance on the working memory updating tests that were trained in the cognitive training (n-back, running span). Specifically, the effect was limited to the composite that captured performance on trained tests that used stimuli that were not seen during the cognitive intervention. In a post-hoc analysis of the individual tests, however, the effect was not only present in the two trained tests with untrained stimuli but also in one of the trained tests with trained stimuli, providing some indication that the effect may be general to performance on the tests that constituted the cognitive training. The effect was also not found for any of the other cognitive composites, suggesting that any effects on training gains did not generalize to performance in other working memory tasks or to other cognitive domains. As such, any advantageous effect appears to be restricted to the specific cognitive tests that coincides with the post-exercise plasma BDNF increase.

Beyond associations with cognitive training outcome, the results provided further support for a positive role of post-exercise increases in plasma BDNF for working memory in general. Specifically, such increases were associated with greater performance on trained working memory tests (trained and untrained stimuli), across timepoints and independent of intervention order, with a similar trend for performance on working memory updating tests that were not trained during the intervention. A trending association was also found between post-exercise increases of plasma BDNF and faster improvements in the non-adaptive updating task that was administered after each cognitive training session, independent of the order of the interventions. Considering that effects were restricted to training progress and to performance on working memory updating tasks, it can be speculated that individuals who demonstrate large increases in plasma BDNF following physical exercise may have an advantage in maintaining and continuously updating information in working memory.

It is interesting to note that exercise-induced increases in serum BDNF had no advantageous effect on training gains in any of the groups or on any of the cognitive composites, across timepoints, which question the relevance of serum BDNF for cognition. The sources and mechanisms behind exercise-induced BDNF changes in serum and plasma BDNF are not well understood^21^. However, considering that platelets are capable of taking up BDNF but not of synthesizing it^19^, it has been proposed that plasma concentrations are more likely to reflect BDNF secreted from the brain^16^. The present results appear consistent with the proposal of plasma BDNF is more relevant for cognitive function than serum BDNF but more research is required.

Considering the demonstrated importance of interindividual differences in the post-exercise BDNF increase in plasma for cognitive training gains, it may seem surprising that no difference in cognitive training outcome was found between the group that received physical exercise before as opposed to after cognitive training. Here it is important to emphasize that in addition to its effects on neurotrophins, physical exercise gives rise to a range of other neurochemical changes in the acute term, including changes in lactate, cortisol, dopamine, norepinephrine, serotonin, GABA and glutamate^47^. Furthermore, factors such as perceived exhaustion, sweating, and general arousal following physical exercise are likely to influence subsequent cognitive performance. Therefore, at the group level, it may be that the any beneficial effect of increased plasma BDNF on subsequent cognition is counteracted by other temporally coupled exercise-induced changes, limiting the overall generalizability of the finding. In other words, whilst a particularly large plasma BDNF increase may allow for a beneficial effect to emerge even in the face of such counteracting effects, the group average increase may not be differential because of the counteracting effects. In this context, it is interesting to note that previous research suggests that greater cardiovascular fitness could be associated with greater acute BDNF increases following physical exercise^12^. Whilst the hypotheses considered here were concerned with acute and not chronic effects of physical exercise on BDNF and cognition, it is possible an improvement in cardiovascular fitness following the exercise intervention in the present study would have had a boosting effect on the acute BDNF increase and thereby its advantageous effect on cognition, even in the face of counteracting factors. Furthermore, regular exercise is likely to give rise to neurochemical and metabolic changes that influence how BDNF concentrations change in response to exercise, both acutely and in the longer-term. A more exhaustive investigation of exercise-induced neurochemical and related changes will therefore be necessary to better understand the role of plasma BDNF and its interacting counterparts for subsequent cognitive engagement. Future research may also consider targeting specific sub-populations that can be expected to exhibit a greater post-exercise BDNF response, such as physically fit individuals, or that may be more susceptible to modulation of this response through regular physical exercise, such as sedentary individuals.

In summary, we provide evidence for a time-critical role of acute changes in peripheral BDNF following physical exercise at an interindividual level, by which individuals with greater increases in plasma BDNF following physical exercise at pretest exhibited greater cognitive training gains, but only if each cognitive training session was preceded as opposed to followed by physical exercise. As such, the results provide important mechanistic insights into how multidomain interventions may be delivered for optimal preventative effects in the future. More research will, however, be required to further elucidate the factors that contribute to and interact with acute changes in plasma BDNF and how such changes more precisely benefits cognitive training outcome in older age.

## Supplementary information

Supplementary information.
