# Supplementary material for: Acute increases in brain-derived neurotrophic factor in plasma following physical exercise relates to subsequent learning in older adults
Source: Sci Rep. 2020 Mar 10;10:4395. doi: 10.1038/s41598-020-60124-0 (PMC7064503; doi:10.1038/s41598-020-60124-0)
Supplement: Supplementary file 1 — Supplementary information. [file 41598_2020_60124_MOESM1_ESM.pdf]

Acute increases in brain-derived neurotrophic factor in plasma following physical exercise relates to subsequent learning in older adults

Jonna Nilsson[1] *, Örjan Ekblom[2], Maria Ekblom[2], Alexander Lebedev[1, 3], Olga Tarassova[2], Marcus Moberg[2] & Martin Lövdén[1]

[1] Aging Research Center, Karolinska Institutet and Stockholm University, Stockholm, Sweden
[2] Swedish School of Sport and Health Sciences, Stockholm, Sweden
[3] Department of Clinical Neuroscience, Karolinska Institutet, Stockholm, Sweden

* Corresponding author
E-mail: jonna.nilsson@ki.se

S1. Complete study criteria. MMSE=Mini Mental State Exam.

**Criteria with relevance for study completion**
Ability to attend all study visits
Ability to maintain current level of physical activity, in addition to any study intervention, during the whole study period
No planned lifestyle changes during the study period (e.g. change of physical exercise routine, diet or leisure activities)
Adequate hearing and normal or corrected eye sight
**Criteria with relevance for good health**
No neurological disease, including all dementia types (MMSE<26 excluded), Parkinson's disease or epilepsy
No current or ongoing cardiovascular disease (blood pressure up to 200/100 included)
No history of brain damage, including stroke
No uncontrolled metabolic disease, including diabetes (untreated type II included) and Grave's disease
No ongoing cancer (<1 year after completed treatment excluded)
No psychiatric illness (history of mild to moderate depression and anxiety included)
No history of head trauma with resulting unconsciousness
**Criteria with relevance for cognitive testing**
Fluency in the Swedish language
No color blindness
No previous participation in the study or in another study where cognitive tests are included
**Criteria with relevance for fitness testing**
No neuromotor or musculoskeletal dysfunction preventing activity on an exercise bike or treadmill
No medications that can affect the fitness tests, such as beta-blockers and beta-stimulants
No ongoing infection
No chest pain
**Criteria with relevance for blood sampling**
No fear of needles
No medication that can influence the blood analysis, including antiepileptic and antidepressant medication, sleeping medication and cortisone treatment

S2. Detailed descriptions of working memory training tasks.

Task	Task description	Difficulty Level	Level description	Criterion (max)
N-back	Stimuli were presented on the screen, one by one, and the task was to press a button whenever the currently shown stimulus was the same as the one presented N steps back in the series. Difficulty was varied by the number of steps (2-back, 3-back) and whether the stimuli were evaluated based on one or two aspects of the stimuli (single, dual). For example, a dual 2-back task required participants to base their decision on the word *and* the color of the stimulus (targets marked with *): sofa, football, chest, football*, tree*, sofa, football, sofa*. The four stimulus sets included words and colours (as in the example), shape and patterns, shape and position in a 3x3 grid, and, number and position in a 3x3 grid. With the exception of position, which included 9 items, stimulus sets consisted of six items. Each stimulus was presented for 2250ms with an interstimulus interval of 250ms. 24 stimuli were presented for each of the eight 1-minute runs with an average of 30% of the stimuli in a run being targets. A fixed criterion, expressed as number correct in a run (hits + correct rejections), had to be reached to move on to the next difficulty level. In each training session, 12 runs were completed. Participants received figural feedback on their performance on each run.	1<br>2<br>3<br>4<br>5<br>6<br>7<br>8<br>9<br>10<br>11<br>12<br>13<br>14<br>15<br>16<br>17<br>18<br>19<br>20<br>21	Single, 2-back<br>Single, 2-back<br>Single, 2-back<br>Single, 2-back<br>Single, 3-back<br>Single, 3-back<br>Single, 3-back<br>Single, 3-back<br>Dual, 2-back<br>Dual, 2-back<br>Dual, 2-back<br>Dual, 2-back<br>Dual, 3-back<br>Dual, 3-back<br>Dual, 3-back<br>Dual, 3-back<br>Dual, 3-back<br>Dual, 3-back<br>Dual, 3-back<br>Dual, 3-back<br>Dual, 3-back	20 (24)<br>20 (24)<br>20 (24)<br>20 (24)<br>20 (24)<br>20 (24)<br>20 (24)<br>20 (24)<br>40 (48)<br>40 (48)<br>40 (48)<br>40 (48)<br>40 (48)<br>40 (48)<br>40 (48)<br>40 (48)<br>41 (48)<br>42 (48)<br>43 (48)<br>44 (48)<br>Repeat
Running span	Stimuli were presented on the screen, one by one, and the task was to remember the N last stimuli. The difficulty was varied by how many stimuli that had to be remembered (2 last, 3 last) and by presenting one or two series of stimuli simultaneously (single, dual). For example, a 2-last dual task required participants to indicate the two last pairs of stimuli whenever the stimuli stream stopped (targets marked with *): money-chair, money-car, ball-chair, elephant*-plane*, elephant*-car* (stimuli presented as images). The four stimulus sets included two image sets (as in the example), letter and shape, word and color, and, number and position in a 3x3 grid. With the exception of position, which included 9 items, stimulus sets consisted of three items. Each stimulus was presented for 2000 ms with an inter-stimulus interval of 250 ms. In each run, a response was required 7 times. At response, all stimuli items were presented and participants responded by clicking the items in the correct order within 8 seconds. A fixed criterion, expressed as number correctly identified stimuli items for each run, had to be reached to move on to the next difficulty level. In each training session, 4 runs were completed. Participants received figural feedback on their performance on each run.	1<br>2<br>3<br>4<br>5<br>6<br>7<br>8<br>9<br>10<br>11<br>12<br>13<br>14<br>15<br>16<br>17<br>18<br>19<br>20<br>21	Single, 2-last<br>Single, 2-last<br>Single, 2-last<br>Single, 2-last<br>Single, 3-last<br>Single, 3-last<br>Single, 3-last<br>Single, 3-last<br>Dual, 2-last<br>Dual, 2-last<br>Dual, 2-last<br>Dual, 2-last<br>Dual, 3-last<br>Dual, 3-last<br>Dual, 3-last<br>Dual, 3-last<br>Dual, 3-last<br>Dual, 3-last<br>Dual, 3-last<br>Dual, 3-last<br>Dual, 3-last	12 (14)<br>12 (14)<br>12 (14)<br>12 (14)<br>18 (21)<br>18 (21)<br>18 (21)<br>18 (21)<br>25 (28)<br>25 (28)<br>25 (28)<br>25 (28)<br>32 (42)<br>32 (42)<br>32 (42)<br>32 (42)<br>34 (42)<br>36 (42)<br>38 (42)<br>40 (42)<br>Repeat

S3. Detailed description of the daily n-back task

Task	Task description
Daily n-back	The daily n-back task was non-adaptive and was administered after each cognitive training session. Stimuli were presented on the screen, one by one, and the task was to press a button whenever the currently shown stimulus was the same as the one presented 3 steps back in the series. Stimuli were evaluated based on a single aspect of the stimuli (identity). The stimuli set consisted of six symbolic pictures that were not part of the cognitive training. Each stimulus was presented for 2250ms with an interstimulus interval of 250ms. A total of 24 stimuli (8 targets) were presented for each of the four 1-minute runs, with 10 seconds break between each run. Subjects received feedback on their performance after each run. Both hits and correct rejections contributing to the score, resulting in a maximum score of 24.

S4. Detailed descriptions of tasks included in the cognitive test battery.

Domain /Composite	Task	Task description	Dependent measure
Trained WM (trained stimuli)	N-back, trained stimuli	See task description for N-back in S3. The test consisted of four runs of single 2-back, four runs of single 3-back and four runs of dual 2-back. The stimuli set consisted of shapes in different positions, which was used during training. In the dual task version, participants were required to base their decision on the shape as well as the position of the stimuli. 288 stimuli were shown, of which 96 were targets (33%). Completion time was approximately 20 minutes.	Number correct (Number hits + number correct rejections)
	Running span, trained stimuli	See task description for Running span in S3. Responses were required at 10 occasions at the 2-last dual level (max correct/trial=4), at 10 occasions at the 3-last single level (max correct/trial=3) and at 10 occasions at the 2-last dual level (max correct/trial=6). The stimuli set consisted of letters and shapes, which was used during training. In the dual task version, participants had to remember the letter as well as the shape. Completion time was approximately 20 minutes.	Average proportion correct per trial
Trained WM (untrained stimuli)	N-back, untrained stimuli	The task was identical to the N-back (trained stimuli) with the exception of the stimuli, which was not used during the cognitive training. The stimuli set consisted of six photographic images (e.g. fire, water) that were presented at different counts (1-6 images). In the dual task version, participants were required to base their decision on the count as well as the identity of the image.	Number correct (Number hits + number correct rejections)
	Running span, untrained stimuli	The task was identical to the Running span task (trained stimuli) with the exception of the stimuli, which was not used during the cognitive training. The stimuli set consisted of two dice presented in different colors showing 1-3 pips, which was not used during training. In the dual task version, participants had to remember the color as well as the number of pips on the dice.	Average proportion correct per trial
Untrained WM (switching)	Task switching 1	Stimuli were presented on the screen, one by one, underneath one out of four one-word questions present on the screen. The stimuli consisted of a circle or a rectangle with a horizontal or a vertical line through it. The four questions were: Circle? Rectangle? Vertical? Horizontal?. For example, when a stimulus (e.g. a circle with a vertical line) appeared underneath the question "Vertical", participants were required to attend to orientation of the line. If the next stimulus was presented underneath a different question, participants were required to switch their attention. Once a response was made the next stimulus was presented. Stimuli were either presented 3-9 times underneath the same question before switching. There were 352 non-switch trials and 62 switch trials. Completion time was approximately 10 minutes.	Switch cost (response time for correct switch trials – response time for correct non-switch trials)

	Task switching 2	The task was identical to Task Switching 1 with the exception of the stimuli. The stimuli consisted of two sets: photographic images of edible or inedible objects that were presented in black-and-white or in color. The four one-word questions were: Black-and-white? Coloured? Edible? Inedible?. There were 351 non-switch trials and 62 switch trials.	Switch cost (response time for correct switch trials – response time for correct non-switch trials)
Untrained WM (updating)	Spatial updating	A display of three 3x3 was shown for 4000 ms in each of which one blue dot was present in one of the nine locations. Those three locations had to be memorized and updated according to shifting operations, which were indicated by arrows appearing below the corresponding field. Presentation time of the arrows was 3000 ms with an inter-stimulus interval of 250 ms. After six updating operations, the three grids reappeared and the resulting three end positions had to be clicked on (maximum three correct responses per trial). Ten trials were completed. Completion time was approximately 10 minutes.	Average proportion correct per trial
	Numerical updating	Four single digits (ranging from 1 to 9) were presented simultaneously in four cells situated horizontally for 3000 ms. After an inter-stimulus interval of 250 ms, a sequence of eight updating operations were presented in a second row of four cells below the first one. These updating operations were additions and subtractions within a range -9 to +9. Each updating operation was applied to a different cell from the one a step earlier in the sequence, so that no two updating operations had to be applied to one cell in a sequence. Those updating operations had to be applied to the digits memorized from the corresponding cells above and the updated results had to be memorized and types in at response (maximum four correct responses per trial). Ten trials were completed. Completion time was approximately 10 minutes.	Average proportion correct per trial
Spatial reasoning	Ravens progressive matrices	The task involved completing as many of the 18 trials within a 12-minute time limit. Each trial consisted of increasingly complex visual geometric designs with a missing piece. The task was to select the missing piece from eight alternatives.	Number correct within time limit
	WASI-II matrices	The task involved completing as many of the 30 trials within a 15-minute time limit. Each trial consisted of increasingly complex visuospatial objects with a missing piece. The task was to select the missing piece from five alternatives.	Number correct within time limit
	BETA-III matrices	The task involved completing as many of the 25 trials within an 8-minute time limit. Each trial consisted of increasingly complex visual geometric designs with a missing piece. The task was to select the missing piece from five alternatives.	Number correct within time limit
Verbal reasoning	ETS kit verbal inference	The task involved completing as many of the 20 verbal inference trials as possible within a 12-minute time limit. The task involved selecting	Number correct within time limit

		the valid conclusion given the information given in a verbal statement. Example trial: One year a particular farmer's stand of wheat yielded 40 bushels per acre. 1) The farmer's land is extremely fertile 2) The farmer has raised wheat on his land 3) The weather that year was unfavourable for growing wheat 4) Forty bushels per acre is a high yield 5) The field would be more suitable for some other crop	
	BIS analogies	The task involved completing as many of the 14 analogies within a 5-minute time limit. The task involved finding completing analogies. Example trial: Newspaper is to text as speaker is to 1) sound 2) song 3) news 4) listening 5) stereo	Number correct within time limit
	Syllogisms	The task involved completing as many of the 30 syllogism trials within a 20-minute time limit. The task on each trial was to decide whether the conclusion that followed the premises was valid or not. Example trial: All trees are fish. All fish are horses. Therefore all trees are horses. 1) valid conclusion 2) invalid conclusion	Number correct within time limit
Episodic memory	Spatial memory	Sequences of 12 colored photographs of real-world objects were displayed at different locations in a 6x6 grid. Each photograph was presented for 8000ms with an inter-stimulus interval of 1000ms. After presentation, objects appeared at the right side of the screen and had to be moved in the correct order to the correct locations by clicking on the objects and the locations with the computer mouse. Two trials were included in the test (maximum number correct=24).	Number correct
	Verbal memory	Sequences of 16 nouns were presented in the middle of the screen. Each noun was presented for 6000ms with an inter-stimulus interval of 1000ms.After presentation, the nouns had to be recalled by typing in the word into a blank field. Two trials were included in the test (maximum number correct=32.	Number correct
Perceptual speed	Perceptual matching 1	Pairs of 4-item letter strings were presented and the task was to decide whether they were identical or not as quickly as possible. The number strings could differ by 1-4 letters. Once a response was made the next stimulus was presented. There were two runs with 50 trials in each run, of which the letter strings differed in 50% of the trials. Participants were instructed to respond as quickly and as accurately as possible. Completion time was approximately 5 minutes.	Average response time for correct trials
	Perceptual matching 2	The task was identical to Perceptual matching 1, with the exception of the stimuli set that consisted of 4-item number strings.	Average response time for correct trials

S5. Descriptive statistics for all cognitive composites at pretest and posttest, separately for each intervention group. Task performance is expressed as T-scores with a mean of 50 and a standard deviation of 10. COG=cognitive training, PE=physical exercise, WM=working memory.

	**COG**			**PE**			**COG+PE**			**PE+COG**		
**Cognitive composite**	n	mean	sd	n	mean	sd	n	mean	sd	n	mean	sd
Trained WM (trained stimuli), pretest	21	51.05	12.28	27	48.82	9.616	24	51.92	9.864	25	48.55	8.517
Trained WM (trained stimuli), posttest	21	64.76	10.82	27	55.49	9.273	24	64.55	9.981	25	62.92	11.10
Trained WM (untrained stimuli), pretest	21	49.01	12.83	27	50.47	9.141	24	51.52	9.318	25	48.87	9.184
Trained WM (untrained stimuli), posttest	21	59.11	11.46	27	52.65	8.332	24	59.90	8.609	25	58.44	12.19
Untrained WM (updating), pretest	21	51.44	9.901	27	48.26	8.790	24	50.34	11.14	25	50.35	10.50
Untrained WM (updating), posttest	21	54.84	10.02	27	50.45	7.472	24	54.92	13.50	25	52.11	10.46
Untrained WM (switching), pretest	21	52.16	10.88	27	52.25	11.62	24	49.42	8.343	25	46.31	7.998
Untrained WM (switching), posttest	21	50.98	10.93	27	52.71	13.00	24	50.84	9.699	25	46.66	7.975
Episodic memory, pretest	21	50.34	10.61	27	52.23	10.59	24	50.74	9.113	25	46.59	9.315
Episodic memory, posttest	20	53.61	10.05	27	53.46	11.23	24	52.93	10.66	24	51.46	9.042
Processing speed, pretest	21	52.14	10.68	27	50.77	7.284	24	49.78	11.35	25	47.57	10.64
Processing speed, posttest	21	54.01	9.791	27	51.30	8.084	24	51.69	9.486	25	49.24	12.50
Spatial reasoning, pretest	21	51.16	10.95	27	49.55	11.16	24	49.47	9.129	25	50.02	9.147
Spatial reasoning, posttest	21	53.42	10.91	26	53.58	9.779	24	53.87	6.801	25	54.31	8.133
Verbal reasoning, pretest	21	51.06	12.21	27	49.75	10.61	24	47.31	8.391	25	51.96	8.604
Verbal reasoning, posttest	21	53.01	12.27	26	50.90	10.11	24	51.49	9.552	25	51.94	9.687

S6. Drop-out analysis comparing age, reasoning ability and self-reported physical activity at pretest for the randomized and the final sample, separately for the four intervention groups. Standardized mean differences (SMD) were calculated as ($M_{rand} - M_{final}$)/SD_{rand}. In absolute terms and considering all intervention groups, SMDs were all small and ranged from 0.06 to 0.15 for age, from 0.004 to 0.05 for reasoning ability, and from 0.03 to 0.14, from 0 to 0.13, from 0.02 to 0.15 for time spent engaging in vigorous, moderate and sedentary activity, respectively.

	**COG**					**PE**				
	Randomized (n=37)		Completers (n=21)			Randomized (n=34)		Completers (n=27)		
	mean	sd	mean	sd	**SMD**	mean	sd	mean	sd	**SMD**
Age (years)	70,68	2,97	70,95	3,04	-0,09	70,12	2,89	70,30	3,00	-0,06
Reasoning ability (score)	5,81	2,73	5,86	2,95	-0,02	6,09	2,75	6,04	2,77	0,02
Vigorous activity (h/week)	3,35	1,57	3,14	1,35	0,13	3,62	1,67	3,78	1,58	-0,10
Moderate activity (h/week)	5,14	1,34	5,14	1,35	0,00	5,21	1,32	5,30	1,30	-0,07
Sedentary activity (h/week)	3,22	0,95	3,24	1,09	-0,02	3,09	0,71	3,07	0,68	0,03
	**COG+PE**					**PE+COG**				
	Randomized (n=35)		Completers (n=24)			Randomized (n=33)		Completers (n=25)		
	mean	sd	mean	sd	**SMD**	mean	sd	mean	sd	**SMD**
Age (years)	70,14	2,92	70,33	3,14	-0,07	69,88	2,68	70,28	2,70	-0,15
Reasoning ability (score)	6,66	3,12	6,50	3,07	0,05	6,15	2,31	6,16	2,48	0,00
Vigorous activity (h/week)	3,40	1,61	3,17	1,69	0,14	3,42	1,87	3,48	1,85	-0,03
Moderate activity (h/week)	5,43	1,17	5,58	1,10	-0,13	5,58	1,28	5,56	1,39	0,02
Sedentary activity (h/week)	3,29	0,79	3,17	0,70	0,15	2,97	0,68	3,00	0,65	-0,04

S7. Descriptive statistics on un-transformed BDNF concentrations from pretest, separately for each intervention group. Sample 1 was taken before the first intervention, sample 2 after the first intervention and sample 3 after the second intervention or after rest, all at pretest. COG=cognitive training, PE=physical exercise.

		**COG**			**PE**			**COG+PE**			**PE+COG**		
		n	*mean*	*sd*	*n*	*mean*	*sd*	*n*	*mean*	*sd*	*n*	*mean*	*sd*
Serum (pg/mL)	Sample 1	20	25011	4958	26	23964	4979	23	25696	7077	25	24198	5336
	Sample 2	20	25238	4510	26	28244	6365	24	26635	6116	25	28314	6803
	Sample 3	20	22730	7890	26	23726	6785	24	29185	7680	25	24616	7358
Plasma (pg/mL)	Sample 1	21	188,5	152	27	389,3	506,6	24	512,1	1358	25	197,9	211,3
	Sample 2	21	1100	1903	27	1463	2045	24	1663	3536	25	683,9	844,5
	Sample 3	21	1358	1730	27	1770	2767	24	2709	3805	25	1270	2025

S8. Unstandardized parameter estimates from the linear mixed effects models and F-tests of main and interaction effects used to test whether cognition in older adults benefits more from repeated sessions of working memory training when each training session is directly preceded as opposed to followed by physical exercise (H1). Linear mixed models with random intercept were employed for all factors, with the exception for cognitive training progress, for which a random linear slope model was employed. Task performance is expressed as T-scores with a mean of 50 and a standard deviation of 10 for all factors, with the exception of cognitive training progress, which is expressed as raw scores (maximum=24). Intervention = COG+PE < PE+COG; Time = pretest < posttest; Training visit = cognitive training visits 1-32; WM=working memory. Note that the corrected α-level was 0.025 for trained WM (trained stimuli, untrained stimuli) and for untrained WM (updating, switching) and 0.0125 for untrained domains (processing speed, episodic memory, spatial reasoning, verbal reasoning).

Hypothesis 1	Estimate	CI (95%)		NumDF	DenDF	F value	Pr(>F)
**Trained WM (trained stimuli)**							
(Intercept)	51,92305	48	55,9				
Intervention	-3,37762	-8,88	2,13	1	47	1,01536	0,31878
Time	12,62595	8,84	16,4	1	47	99,3771	3,54E-13
Intervention x Time	1,744515	-3,56	7,05	1	47	0,41498	0,52259
**Trained WM (untrained stimuli)**							
(Intercept)	51,51636	47,6	55,5				
Intervention	-2,64697	-8,19	2,9	1	47	0,58753	0,44721
Time	8,388224	5,77	11	1	47	92,0273	1,20E-12
Intervention x Time	1,180867	-2,48	4,85	1	47	0,39796	0,5312
**Untrained WM (updating)**							
(Intercept)	50,34	45,78	54,90				
Intervention	0,01	-6,37	6,40	1,00	47,00	0,20	0,66
Time	4,59	1,90	7,28	1,00	47,00	10,86	0,00
Intervention x Time	-2,83	-6,60	0,94	1,00	47,00	2,16	0,15
**Untrained WM (switching)**							
(Intercept)	49,42	46,03	52,82				
Intervention	-3,11	-7,86	1,64	1,00	47,00	2,54	0,12
Time	1,42	-0,89	3,73	1,00	47,00	1,14	0,29
Intervention x Time	-1,07	-4,31	2,17	1,00	47,00	0,42	0,52
**Episodic memory**							
(Intercept)	50,74	46,94	54,55				
Intervention	-4,15	-9,48	1,18	1,00	46,92	1,06	0,31
Time	2,19	-0,46	4,85	1,00	46,15	14,87	0,00
Intervention x Time	3,00	-0,75	6,75	1,00	46,15	2,46	0,12
**Processing speed**							
(Intercept)	49,78	45,37	54,20				
Intervention	-2,21	-8,39	3,97	1,00	47,00	0,57	0,45
Time	1,90	-0,07	3,88	1,00	47,00	6,41	0,01

Intervention x Time	-0,24	-3,00	2,52	1,00	47,00	0,03	0,87
**Spatial reasoning**							
(Intercept)	49,47	46,15	52,80				
Intervention	0,55	-4,11	5,21	1,00	47,00	0,05	0,83
Time	4,40	1,91	6,89	1,00	47,00	23,78	0,00
Intervention x Time	-0,12	-3,60	3,37	1,00	47,00	0,00	0,95
**Verbal reasoning**							
(Intercept)	47,31	43,70	50,92				
Intervention	4,65	-0,40	9,71	1,00	47,00	1,12	0,29
Time	4,18	1,48	6,89	1,00	47,00	4,63	0,04
Intervention x Time	-4,20	-7,99	-0,41	1,00	47,00	4,71	0,04
**Cognitive training progress**							
(Intercept)	17,24321	16,8	17,7				
Intervention	0,056825	0,04	0,08	1	46,428	74,9066	3,02E-11
Time	-0,03583	-0,72	0,65	1	46,988	0,01037	0,91934
Intervention x Time	0,009803	-0,02	0,04	1	46,428	0,4723	0,49535

S9. Unstandardized parameter estimates from the linear mixed effects models and F-tests of main and interaction effects used to test whether cognition in older adults benefits more from repeated sessions of working memory training when each training session is combined with physical exercise, irrespective of order, compared to working memory training alone (H3). Linear mixed models with random intercept were employed for all factors, with the exception for cognitive training progress, for which a random linear slope model was employed. Task performance is expressed as T-scores with a mean of 50 and a standard deviation of 10 for all factors, with the exception of cognitive training progress, which is expressed as raw scores (maximum=24). Intervention = COG < COMB; COMB=PE+COG & COG+PE; Time = pretest < posttest; Training visit = cognitive training visits 1-32; WM=working memory. Note that the corrected α-level was 0.025 for trained WM (trained stimuli, untrained stimuli) and for untrained WM (updating, switching) and 0.0125 for untrained domains (processing speed, episodic memory, spatial reasoning, verbal reasoning).

Hypothesis 3	Estimate	CI (95%)		NumDF	DenDF	F value	Pr(>F)
**Trained WM (trained stimuli)**							
(Intercept)	51,05	46,61	55,48				
Intervention	-0,85	-6,14	4,45	1,00	68,00	0,15	0,70
Time	13,71	9,96	17,47	1,00	68,00	141,28	<0.001
Intervention x Time	-0,20	-4,68	4,29	1,00	68,00	0,01	0,93
**Trained WM (untrained stimuli)**							
(Intercept)	49,01	44,48	53,54				
Intervention	1,16	-4,25	6,57	1,00	68,00	0,05	0,82
Time	10,10	7,53	12,68	1,00	68,00	147,70	<0.001
Intervention x Time	-1,11	-4,19	1,96	1,00	68,00	0,50	0,48
**Untrained WM (updating)**							
(Intercept)	51,44	46,76	56,12				
Intervention	-1,09	-6,69	4,50	1,00	68,00	0,20	0,66
Time	3,40	0,52	6,27	1,00	68,00	13,91	<0.001
Intervention x Time	-0,26	-3,69	3,18	1,00	68,00	0,02	0,88
**Untrained WM (switching)**							
(Intercept)	52,16	48,17	56,15				
Intervention	-4,33	-9,10	0,45	1,00	68,00	1,99	0,16
Time	-1,18	-3,50	1,13	1,00	68,00	0,05	0,83
Intervention x Time	2,05	-0,71	4,82	1,00	68,00	2,11	0,15
**Episodic memory**							
(Intercept)	50,34	46,17	54,51				
Intervention	-1,71	-6,70	3,27	1,00	68,44	0,37	0,54
Time	3,13	-0,29	6,56	1,00	67,05	10,73	<0.001
Intervention x Time	0,56	-3,53	4,63	1,00	67,05	0,07	0,79
**Processing speed**							
(Intercept)	52,14	47,54	56,74				
Intervention	-3,48	-8,99	2,02	1,00	68,00	1,68	0,20

Time	1,86	-0,50	4,23	1,00	68,00	6,38	0,01
Intervention x Time	-0,08	-2,91	2,75	1,00	68,00	0,00	0,96
**Spatial reasoning**							
(Intercept)	51,16	47,26	55,06				
Intervention	-1,41	-6,06	3,25	1,00	68,00	0,03	0,87
Time	2,26	-0,28	4,79	1,00	68,00	18,19	<0.001
Intervention x Time	2,09	-0,94	5,12	1,00	68,00	1,82	0,18
**Verbal reasoning**							
(Intercept)	51,06	46,73	55,38				
Intervention	-1,37	-6,54	3,80	1,00	68,00	0,28	0,60
Time	1,95	-0,85	4,76	1,00	68,00	5,44	0,02
Intervention x Time	0,09	-3,27	3,44	1,00	68,00	0,00	0,96
**Cognitive training progress**							
(Intercept)	17,23	16,69	17,76				
Training visit	0,08	0,06	0,10	1,00	66,16	111,35	<0.001
Intervention	0,00	-0,64	0,64	1,00	68,13	0,00	1,00
Intervention x Training visit	-0,02	-0,04	0,01	1,00	66,16	1,81	0,18

S10. Unstandardized parameter estimates from the linear mixed effects models and F-tests of main and interaction effects used to test whether cognition in older adults benefits more from repeated sessions of physical exercise when each exercise session is combined with working memory training, irrespective of order, compared to physical exercise alone (H4). Linear mixed models with random intercept were employed for all factors. Task performance is expressed as T-scores with a mean of 50 and a standard deviation of 10. Intervention = PE < COMB; COMB=PE+COG & COG+PE; Time = pretest < posttest; Training visit = cognitive training visits 1-32; WM=working memory. Note that the corrected α-level was 0.025 for trained WM (trained stimuli, untrained stimuli) and for untrained WM (updating, switching) and 0.0125 for untrained domains (processing speed, episodic memory, spatial reasoning, verbal reasoning).

Hypothesis 4	Estimate	CI (95%)		NumDF	DenDF	F value	Pr(>F)
**Trained WM (trained stimuli)**							
(Intercept)	50,20	47,48	52,92				
Intervention	-1,38	-5,93	3,18	1,00	74,00	5,09	0,03
Time	13,52	11,28	15,75	1,00	74,00	110,80	<0.001
Intervention x Time	-6,85	-10,61	-3,09	1,00	74,00	12,76	<0.001
**Trained WM (untrained stimuli)**							
(Intercept)	50,17	47,51	52,82				
Intervention	0,30	-4,15	4,76	1,00	74,00	2,05	0,16
Time	8,99	7,31	10,67	1,00	74,00	60,22	<0.001
Intervention x Time	-6,81	-9,63	-3,99	1,00	74,00	22,37	<0.001
**Untrained WM (updating)**							
(Intercept)	50,34	47,45	53,24				
Intervention	-2,09	-6,94	2,76	1,00	74,00	1,20	0,28
Time	3,14	1,22	5,06	1,00	74,00	10,55	<0.001
Intervention x Time	-0,95	-4,17	2,27	1,00	74,00	0,33	0,57
**Untrained WM (switching)**							
(Intercept)	47,84	45,02	50,65				
Intervention	4,41	-0,32	9,14	1,00	74,00	3,24	0,08
Time	0,87	-0,58	2,32	1,00	74,00	1,15	0,29
Intervention x Time	-0,41	-2,85	2,02	1,00	74,00	0,11	0,74
**Episodic memory**							
(Intercept)	48,63	45,82	51,44				
Intervention	3,60	-1,11	8,32	1,00	73,94	1,10	0,30
Time	3,70	1,65	5,75	1,00	73,20	7,96	0,01
Intervention x Time	-2,47	-5,89	0,96	1,00	73,20	2,00	0,16
**Processing speed**							
(Intercept)	48,66	45,87	51,44				
Intervention	2,12	-2,56	6,79	1,00	74,00	0,42	0,52
Time	1,78	0,33	3,23	1,00	74,00	3,46	0,07
Intervention x Time	-1,25	-3,69	1,18	1,00	74,00	1,02	0,32

**Spatial reasoning**							
(Intercept)	49,75	47,21	52,29				
Intervention	-0,21	-4,47	4,05	1,00	74,34	0,03	0,87
Time	4,34	2,64	6,05	1,00	73,55	32,68	<0.001
Intervention x Time	-0,24	-3,13	2,65	1,00	73,55	0,03	0,87
**Verbal reasoning**							
(Intercept)	49,68	47,00	52,36				
Intervention	0,07	-4,42	4,57	1,00	74,02	0,01	0,93
Time	2,04	0,24	3,84	1,00	73,24	5,17	0,03
Intervention x Time	-0,53	-3,59	2,51	1,00	73,24	0,12	0,73

S11. Unstandardized parameter estimates from the linear mixed effects models and F-tests of main and interaction effects used to test whether the relationship between change in peripheral BDNF levels in response to physical exercise at pretest and the outcome of repeated sessions of working memory training is greater when each training session is directly preceded as opposed to followed by physical exercise (H2). Linear mixed models with random intercept were employed for all factors, with the exception for cognitive training progress, for which a random linear slope model was employed. Task performance is expressed as T-scores with a mean of 50 and a standard deviation of 10 for all factors, with the exception of cognitive training progress, which is expressed as raw scores (maximum=24). Separate analyses were performed for serum and plasma. Intervention = COG+PE < PE+COG; Time = pretest < posttest; Training visit = cognitive training visits 1-32; BDNF=acute concentration change following physical exercise at pretest; WM=working memory. Note that the corrected alpha-level was 0.0125 for trained WM (trained stimuli, untrained stimuli) and for untrained WM (updating, switching), 0.00625 for untrained domains (processing speed, episodic memory, spatial reasoning, verbal reasoning) and 0.025 for cognitive training progress.

Hypothesis 2	Estimate	CI (95%)		NumDF	DenDF	F value	Pr(>F)
**Serum**							
**Untrained WM (updating)**							
(Intercept)	51,62976	50,259	58,543				
Intervention	1,10522	-6,121	5,593	1,00	45,00	0,007	0,932
Time	5,91545	1,328	4,947	1,00	45,00	11,032	0,002
BDNF	-0,00051	-1,578	0,086	1,00	45,00	2,952	0,093
Intervention x Time	-2,95628	-4,037	1,081	1,00	45,00	1,224	0,274
Intervention x BDNF	-0,00007	-1,147	1,206	1,00	45,00	0,002	0,962
Time x BDNF	-0,00052	-0,651	0,076	1,00	45,00	2,299	0,136
Intervention x Time x BDNF	0,00023	-0,400	0,629	1,00	45,00	0,182	0,672
**Untrained WM (switching)**							
(Intercept)	48,00476	43,778	49,894				
Intervention	-4,33594	-6,990	1,660	1,00	45,00	1,393	0,244
Time	1,43053	-0,144	2,969	1,00	45,00	3,024	0,089
BDNF	0,00056	-0,151	1,078	1,00	45,00	2,087	0,156
Intervention x Time	1,13495	-1,634	2,769	1,00	45,00	0,244	0,624
Intervention x BDNF	0,00009	-0,998	0,740	1,00	45,00	0,080	0,778
Time x BDNF	0,00000	-0,505	0,120	1,00	45,00	1,387	0,245
Intervention x Time x BDNF	-0,00053	-0,709	0,175	1,00	45,00	1,340	0,253
**Episodic memory**							
(Intercept)	50,13981	45,061	51,870				
Intervention	-7,02965	-8,383	1,246	1,00	44,90	2,014	0,163
Time	1,69722	0,774	4,436	1,00	44,15	7,415	0,009
BDNF	0,00024	-0,123	1,248	1,00	45,27	2,467	0,123
Intervention x Time	3,96969	-0,603	4,576	1,00	44,15	2,155	0,149
Intervention x BDNF	0,00061	-0,645	1,294	1,00	45,27	0,409	0,526
Time x BDNF	0,00019	-0,345	0,402	1,00	44,50	0,022	0,882

Intervention x Time x BDNF	-0,00030	-0,682	0,375	1,00	44,50	0,305	0,584
**Processing speed**							
(Intercept)	48,25636	48,622	54,687				
Intervention	-5,13065	-4,081	4,495	1,00	45,00	1,337	0,254
Time	1,95158	1,875	5,240	1,00	45,00	4,753	0,035
BDNF	0,00060	-0,533	0,686	1,00	45,00	3,442	0,070
Intervention x Time	0,46821	-1,147	3,611	1,00	45,00	0,055	0,816
Intervention x BDNF	0,00048	-0,845	0,878	1,00	45,00	0,220	0,641
Time x BDNF	-0,00002	-0,424	0,251	1,00	45,00	0,251	0,619
Intervention x Time x BDNF	-0,00016	-0,836	0,120	1,00	45,00	0,167	0,684
**Spatial reasoning**							
(Intercept)	49,60856	42,727	50,841				
Intervention	-0,93876	-9,200	2,275	1,00	45,00	0,009	0,927
Time	3,79906	0,188	2,904	1,00	45,00	16,414	<0.0001
BDNF	-0,00005	-0,026	1,604	1,00	45,00	0,058	0,811
Intervention x Time	2,46388	-1,686	2,155	1,00	45,00	0,984	0,326
Intervention x BDNF	0,00038	-0,870	1,435	1,00	45,00	0,001	0,971
Time x BDNF	0,00024	-0,344	0,201	1,00	45,00	0,240	0,626
Intervention x Time x BDNF	-0,00072	-0,468	0,303	1,00	45,00	2,061	0,158
**Verbal reasoning**							
(Intercept)	45,89749	45,769	52,207				
Intervention	3,34320	-3,702	5,402	1,00	45,00	0,128	0,722
Time	4,97946	0,154	3,860	1,00	45,00	4,304	0,044
BDNF	0,00055	-0,161	1,132	1,00	45,00	2,070	0,157
Intervention x Time	-4,28334	-4,762	0,479	1,00	45,00	2,451	0,124
Intervention x BDNF	0,00011	-0,789	1,040	1,00	45,00	0,069	0,794
Time x BDNF	-0,00031	-0,544	0,200	1,00	45,00	0,783	0,381
Intervention x Time x BDNF	0,00014	-0,457	0,596	1,00	45,00	0,065	0,800
**Cognitive training progress**							
(Intercept)	17,52126	16,950	18,093				
Training visit	0,06358	0,040	0,088	1,00	44,65	40,375	<0.0001
BDNF	-0,10900	-0,235	0,017	1,00	44,95	0,103	0,750
Intervention	-0,63433	-1,558	0,288	1,00	45,03	1,732	0,195
Training visit x BDNF	-0,00265	-0,008	0,003	1,00	44,20	0,276	0,602
Training visit x Intervention	0,00085	-0,038	0,039	1,00	44,65	0,002	0,967
BDNF x Intervention	0,18692	0,002	0,373	1,00	44,95	3,730	0,060
Intervention x Training x BDNF	0,00318	-0,005	0,011	1,00	44,20	0,620	0,435
**Plasma**							
**Untrained WM (updating)**							
(Intercept)	48,91945	45,069	52,770				
Intervention	-1,50232	-6,948	3,944	1,00	45,00	0,279	0,600
Time	2,14866	0,385	3,912	1,00	45,00	5,450	0,024
BDNF	3,25095	0,476	6,026	1,00	45,00	5,038	0,030

Intervention x Time	-0,94239	-3,436	1,551	1,00	45,00	0,524	0,473
Intervention x BDNF	0,08684	-3,837	4,011	1,00	45,00	0,002	0,966
Time x BDNF	0,13889	-1,132	1,409	1,00	45,00	0,044	0,835
Intervention x Time x BDNF	-0,53141	-2,328	1,265	1,00	45,00	0,321	0,574
**Untrained WM (switching)**							
(Intercept)	47,46754	44,580	50,356				
Intervention	-4,70817	-8,792	-0,624	1,00	45,00	4,877	0,032
Time	0,45401	-1,059	1,967	1,00	45,00	0,330	0,568
BDNF	0,75325	-1,328	2,834	1,00	45,00	0,481	0,492
Intervention x Time	-0,08034	-2,220	2,059	1,00	45,00	0,005	0,943
Intervention x BDNF	2,19420	-0,749	5,137	1,00	45,00	2,040	0,160
Time x BDNF	0,22157	-0,869	1,312	1,00	45,00	0,152	0,699
Intervention x Time x BDNF	-0,52491	-2,067	1,017	1,00	45,00	0,425	0,518
**Episodic memory**							
(Intercept)	49,52261	46,217	52,832				
Intervention	-1,60941	-6,284	3,071	1,00	44,88	0,434	0,513
Time	1,49019	-0,150	3,124	1,00	44,09	3,038	0,088
BDNF	1,10763	-1,274	3,488	1,00	44,74	0,794	0,378
Intervention x Time	2,74566	0,426	5,057	1,00	44,09	5,157	0,028
Intervention x BDNF	-0,43966	-3,808	2,927	1,00	44,74	0,063	0,804
Time x BDNF	1,32515	0,154	2,497	1,00	43,96	4,691	0,036
Intervention x Time x BDNF	-1,53196	-3,188	0,126	1,00	43,96	3,135	0,084
**Processing speed**							
(Intercept)	51,98944	47,774	53,416				
Intervention	1,13296	-3,074	4,905	1,00	45,00	0,165	0,687
Time	1,40573	2,415	5,567	1,00	45,00	4,447	0,041
BDNF	-2,42595	-0,541	3,524	1,00	45,00	2,906	0,095
Intervention x Time	0,60339	-1,554	2,903	1,00	45,00	0,410	0,525
Intervention x BDNF	-2,66089	-3,703	2,046	1,00	45,00	1,748	0,193
Time x BDNF	-0,10041	-2,084	0,187	1,00	45,00	0,044	0,835
Intervention x Time x BDNF	-0,76783	-2,262	0,949	1,00	45,00	1,278	0,264
**Spatial reasoning**							
(Intercept)	50,59493	48,206	55,773				
Intervention	0,91513	-4,217	6,483	1,00	45,00	0,193	0,662
Time	3,99083	0,129	2,683	1,00	45,00	23,537	<0.0001
BDNF	1,49157	-5,152	0,300	1,00	45,00	1,976	0,167
Intervention x Time	0,67468	-1,203	2,409	1,00	45,00	0,336	0,565
Intervention x BDNF	-0,82880	-6,516	1,194	1,00	45,00	0,305	0,583
Time x BDNF	-0,94837	-1,021	0,820	1,00	45,00	2,560	0,117
Intervention x Time x BDNF	-0,65646	-2,069	0,533	1,00	45,00	0,613	0,438
**Verbal reasoning**							
(Intercept)	49,76721	46,660	52,874				
Intervention	1,82372	-2,570	6,217	1,00	45,00	0,632	0,431

Time	2,03843	0,277	3,800	1,00	45,00	4,914	0,032
BDNF	0,99514	-1,243	3,234	1,00	45,00	0,725	0,399
Intervention x Time	-2,23835	-4,730	0,253	1,00	45,00	2,962	0,092
Intervention x BDNF	-0,16351	-3,329	3,002	1,00	45,00	0,010	0,922
Time x BDNF	-0,62994	-1,899	0,639	1,00	45,00	0,904	0,347
Intervention x Time x BDNF	0,24090	-1,554	2,036	1,00	45,00	0,066	0,798
**Cognitive training progress**							
(Intercept)	16,97981	16,356	17,602				
Training visit	0,04462	0,020	0,069	1,00	43,80	27,792	0,000
BDNF	0,31507	-0,170	0,800	1,00	44,86	2,923	0,094
Intervention	-0,03256	-0,918	0,854	1,00	44,81	0,005	0,944
Training visit x BDNF	0,01491	-0,004	0,034	1,00	44,15	5,068	0,029
Training visit x Intervention	0,00706	-0,028	0,042	1,00	43,80	0,149	0,701
BDNF x Intervention	-0,06003	-0,698	0,579	1,00	44,86	0,032	0,858
Intervention x Training x BDNF	-0,00015	-0,025	0,025	1,00	44,15	0,000	0,991

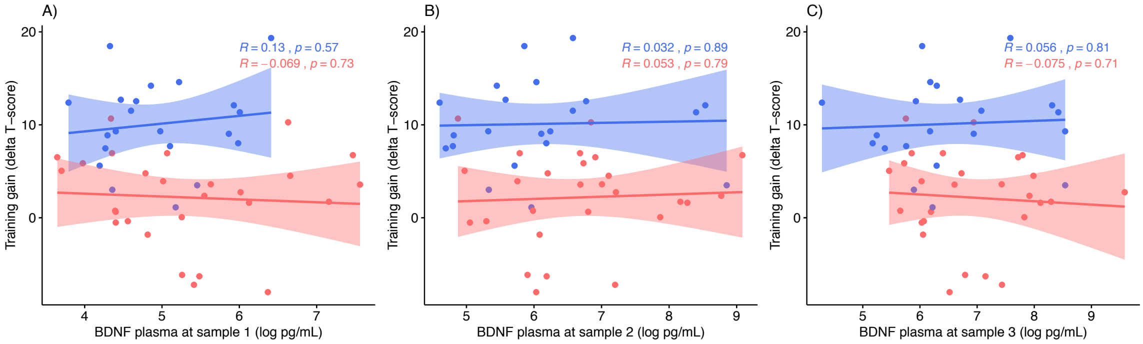

S12. Scatterplots visualizing individual data points and zero-order correlations between training gains (pre-post change in performance on trained tasks with untrained stimuli) and BDNF concentrations in sample 1 (A), sample 2 (B) and sample 3 (C) at pretest, separate for the group that received physical exercise only during the intervention (PE, in red) and the group that received cognitive training only during the intervention (COG, in blue).
